# Supplementary material for: Whole-genome characterization of Plasmodium falciparum population structure and drug resistance across Uganda and neighboring countries
Source: bioRxiv. 2026 Sep 30:2026.09.27.754784. Preprint. [Version 1] doi: 10.64898/2026.09.27.754784 (PMC13648498; doi:10.64898/2026.09.27.754784)
Supplement: Supplementary file 1 [file media-1.pdf]

Whole-genome characterization of *Plasmodium falciparum* population structure and drug resistance across Uganda and neighboring countries

Shahiid Kiyaga[1,2,3,4*], Monica Mbabazi[1,2], Thomas Katairo[2,5,6], Victor Asua[2,7], Francis D. Semakuba[2,5], Bienvenu Nsengimaana[2,5], Stephen Tukwasibwe[2,5,8,9], Innocent Wiringilimaana[2,5], Jackie Nakasaanya[2,5], Jerry Mulondo[2], Eric Watyekele[2], Catherine Maiteki-Sebuguzi[10], David Patrick Kateete[1], Joyce Nakatumba Nabende[11], Daudi Jjingo[3,4,11], Gerald Mboowa[3,12], Charles Batte[13], Samuel L. Nsobya[2], Moses R. Kamya[2,14], Bosco B. Agaba[10,15] , Isaac Ssewanyana[2,5], Andrés Aranda-Diaz[16], Fehintola Victoria Ajogbasile[17], Milya Davlieva[17], Grant Dorsey[17], Philip J. Rosenthal[17], Melissa D. Conrad[18], Bryan Greenhouse[17], Nicholas Hathaway[17,*,+], and Jessica Briggs[17,*,+]

Affiliations

[1]Department of Immunology and Molecular Biology, Makerere University College of Health Sciences, Kampala, Uganda

[2]Infectious Diseases Research Collaboration, Kampala, Uganda

[3]The African Center of Excellence in Bioinformatics and Data Intensive Sciences, Kampala, Uganda

[4]The Infectious Diseases Institute, Makerere University, Kampala, Uganda

[5]National Malaria Reference and Research Laboratory (NMRRL), Central Public Health Laboratories (CPHL), Kampala, Uganda.

[6]Liverpool School of Tropical Medicine (LSTM), Liverpool, UK

[7]Institute for Tropical Medicine, University of Tübingen, Tübingen, Germany

[8]Department of Medical Biochemistry, Makerere University College of Health Sciences, Kampala, Uganda

[9]Uganda Christian University, Mukono, Uganda

[10]National Malaria Elimination Division, Ministry of Health Uganda, Kampala, Uganda

[11]Department of Computer Science, College of Computing and Information Sciences, Makerere University (CoCIS), Kampala, Uganda

[12]Broad Institute of MIT and Harvard, Cambridge, MA, USA

[13]Lung Institute, Makerere University College of Health Sciences, Kampala, Uganda

[14]Department of Medicine, Makerere University College of Health Sciences, Kampala, Uganda

[15]Department of Medical Laboratory Science, Faculty of Medicine, Mbarara University of Science and Technology, Mbarara, Uganda

[16]Barcelona Institute for Global Health (ISGlobal), Barcelona, Spain

[17]Division of HIV, Infectious Diseases and Global Medicine, Department of Medicine, University of California, San Francisco, San Francisco, CA, USA

[18]Johns Hopkins Malaria Research Institute, Department of Molecular Microbiology and Immunology, Bloomberg School of Public Health, Johns Hopkins University, Baltimore, USA

[+]Co-last authors

[*]*Co-corresponding authors:* Shahiid Kiyaga (ashakykiyaga91@gmail.com)

Nicholas Hathaway (Nicholas.Hathaway@ucsf.edu)

Jessica Briggs (Jessica.Briggs@ucsf.edu)

Keywords: *Plasmodium falciparum*; Uganda; whole-genome sequencing; population structure; genetic diversity; identity-by-descent; Selective sweeps; Artemisinin resistance

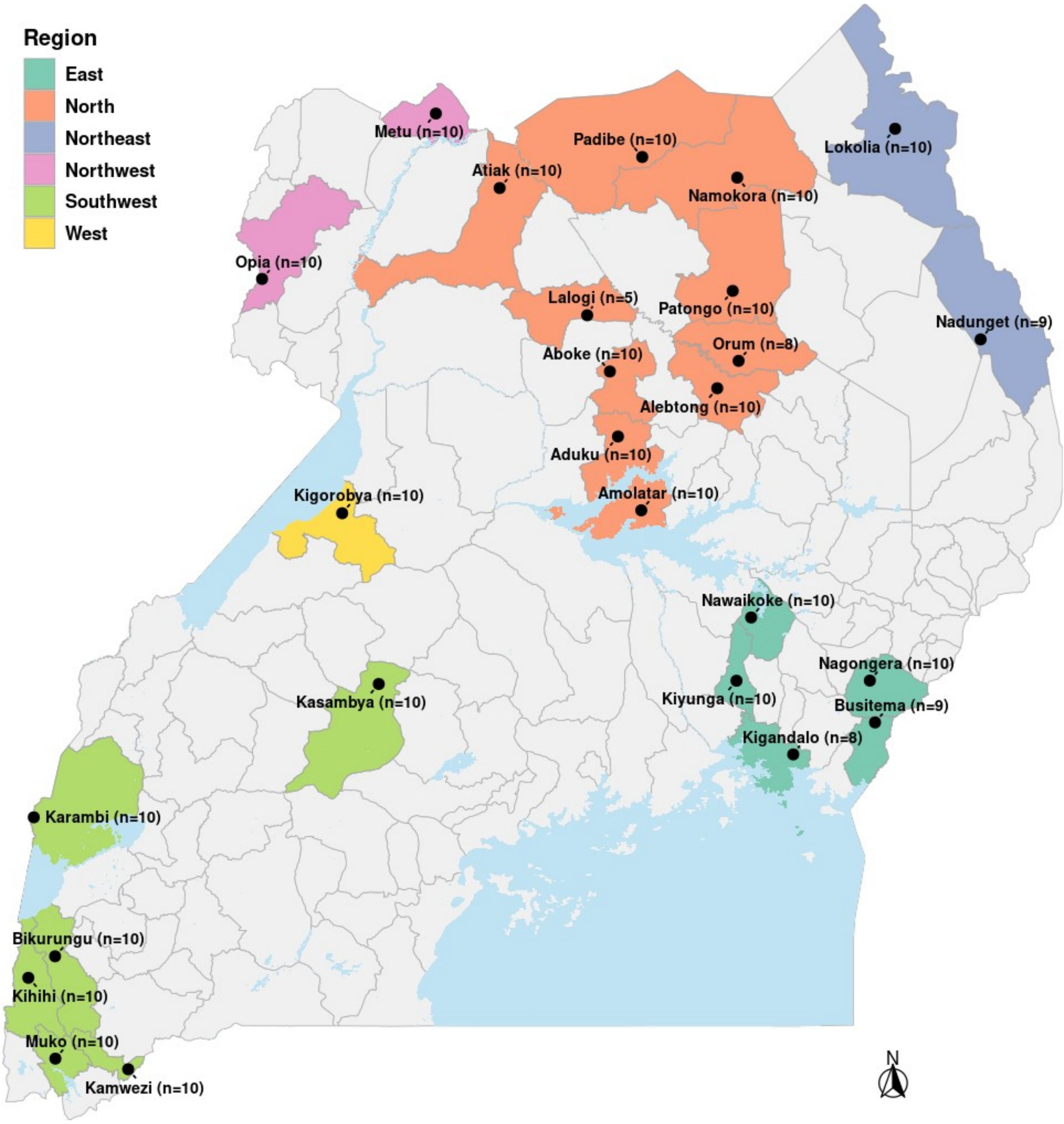

Supplementary Figure 1. Map of sentinel sites by region with sample counts. Geographic distribution of the 26 sentinel surveillance sites across Uganda's six epidemiological regions (Southwest, East, Northeast, North, Northwest, and West). Regions are represented by colors. Annotation in brackets at the end of each site name indicates the number of *P. falciparum* whole-genome sequences successfully generated from each site.

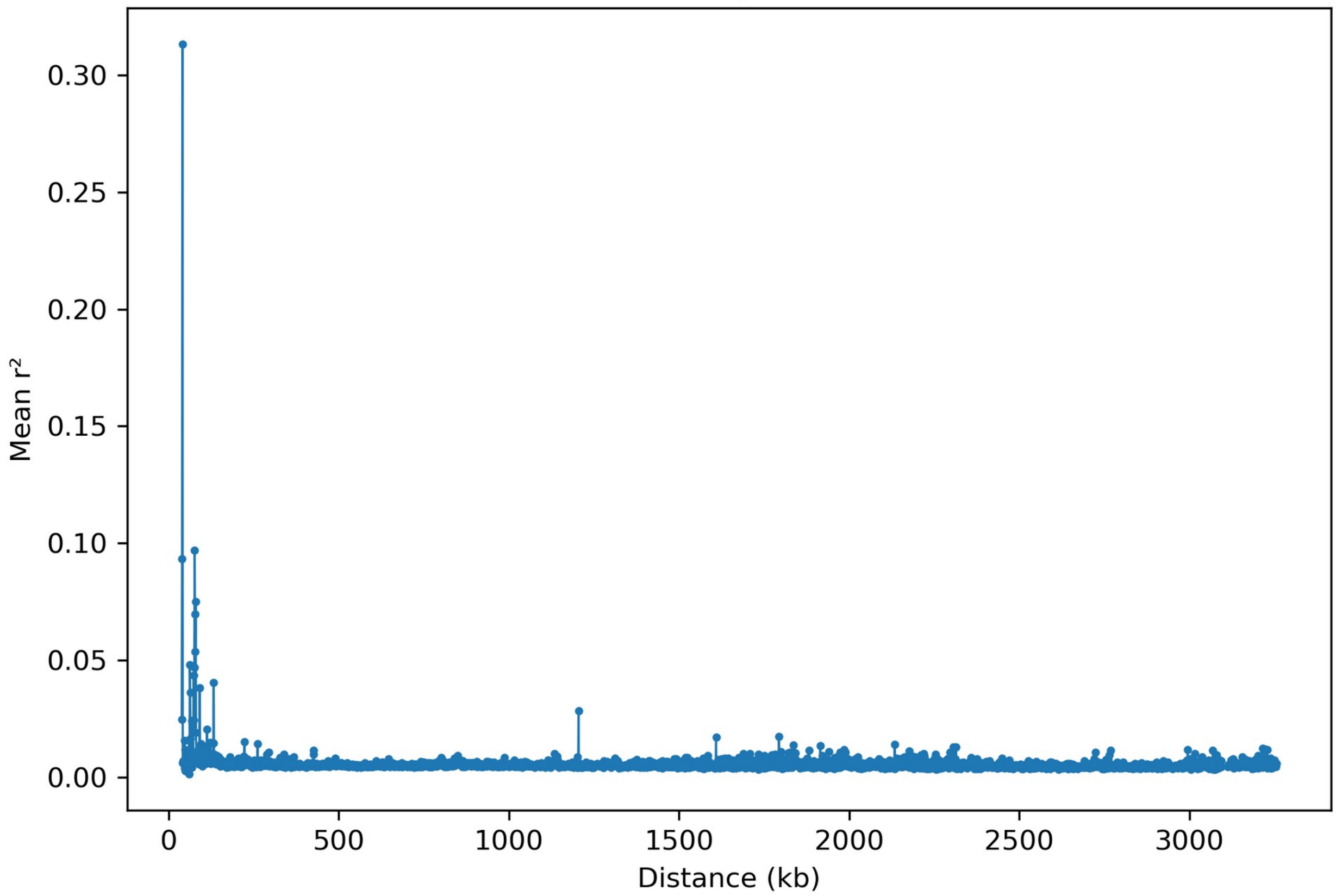

Supplementary Figure 2. Genome-wide LD decay curve (all samples). The curve illustrates the rate at which statistical association between alleles decays with physical distance, providing a baseline measure of recombination and population structure across the full Ugandan dataset.

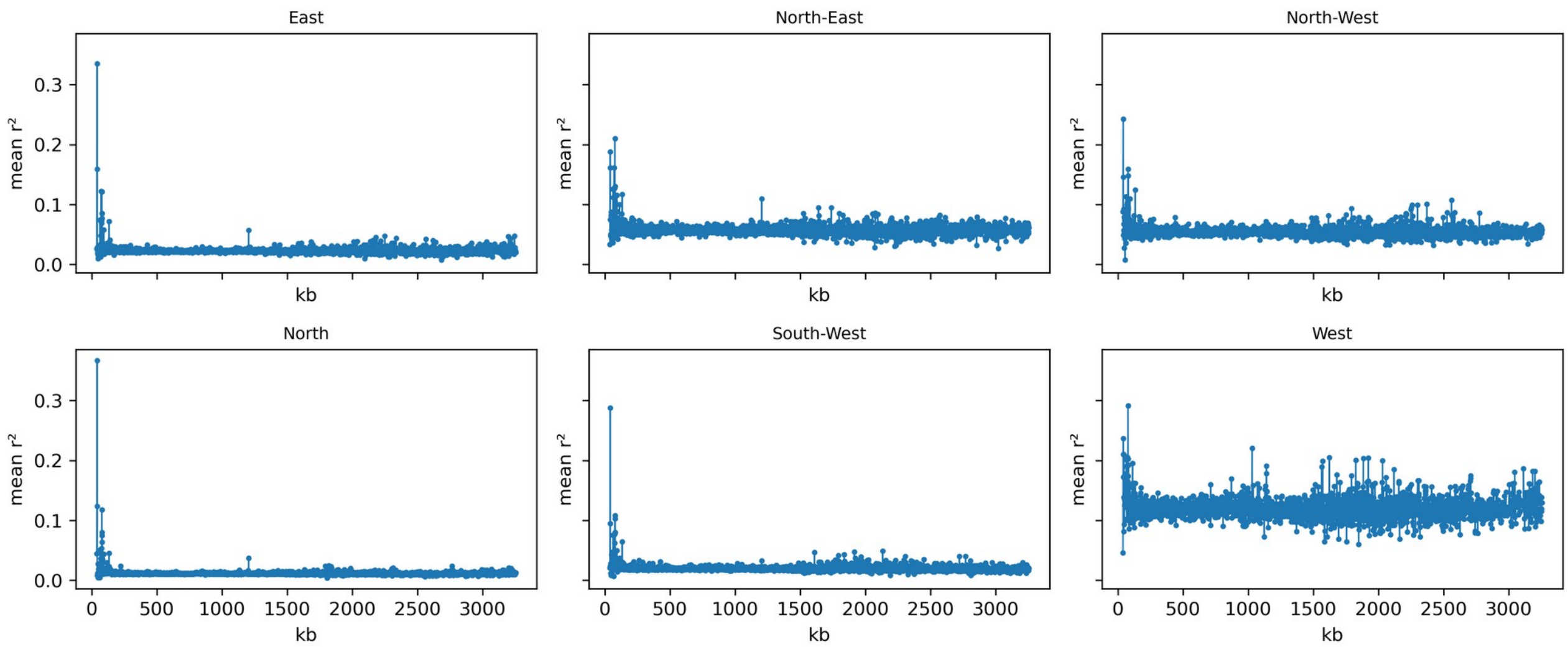

Supplementary Figure 3. LD decay curves faceted by region. Region-specific LD decay profiles (r^2 vs. distance in kb) are shown separately for each of the six geographic regions. Differences in the shape or rate of decay between regions may reflect variation in local effective population sizes, transmission intensity, and extent of inbreeding.

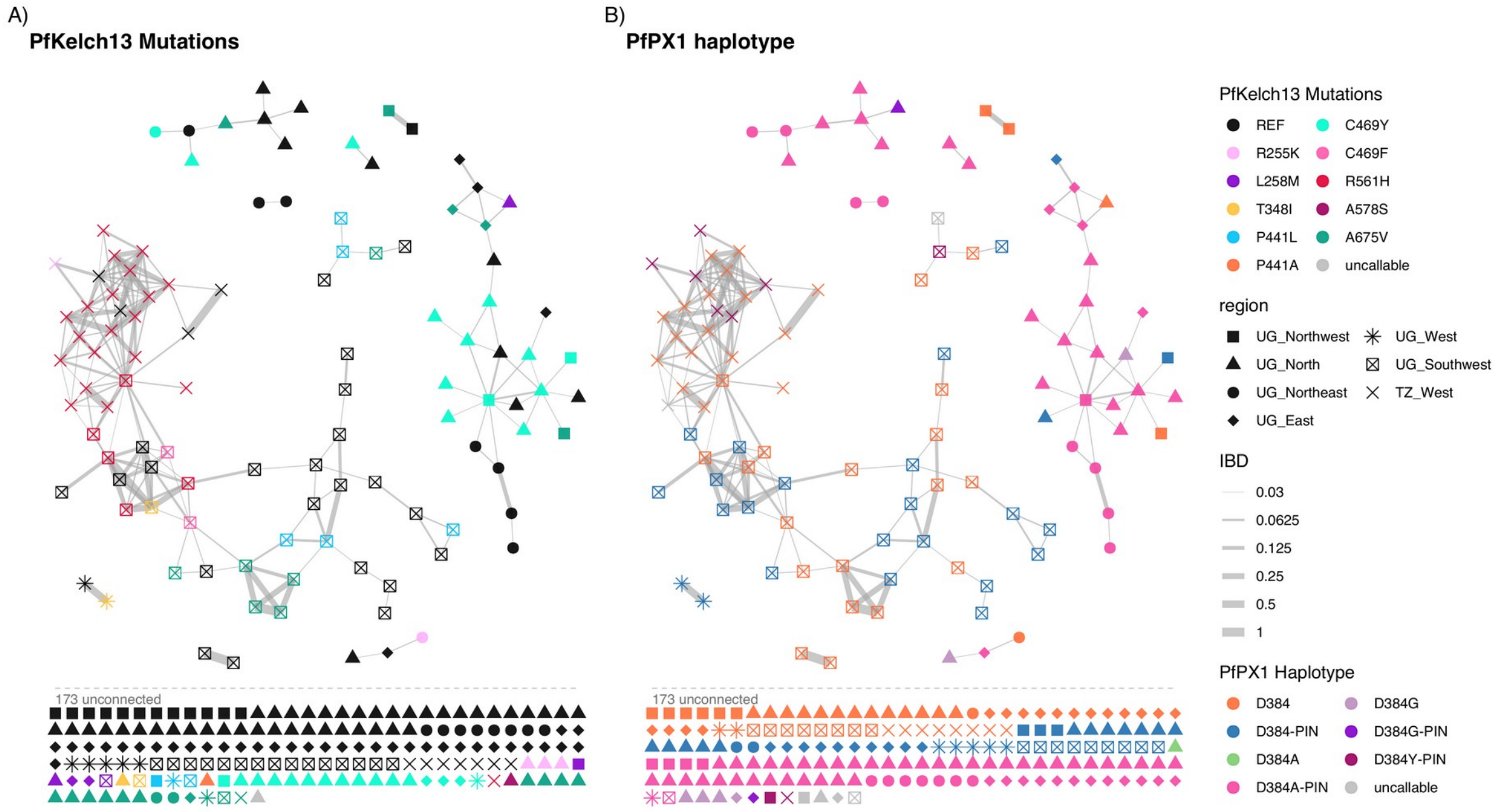

Supplementary Figure 4. Sample-level IBD network coloured by PfKelch13 genotype, PfPX1 haplotype and region. Each node represents an individual *P. falciparum* isolate; edges connect pairs sharing IBD >= 0.03125 (1/32). Node colour indicates the PfKelch13 genotype (wild-type vs. mutant in A), and in b) the PfPX1 haplotype (D384 variant with or without the PIN haplotype), and node shape indicates the sample's geographic region, enabling assessment of whether resistance variants cluster within or spread across regional transmission networks. The majority of the connections between the samples in the Northern regions are between isolates containing the 384A-PIN variant, are under a recent selection sweep which likely contributes to the amount of sharing being seen in those regions rather than sharing due to geographical constraint.

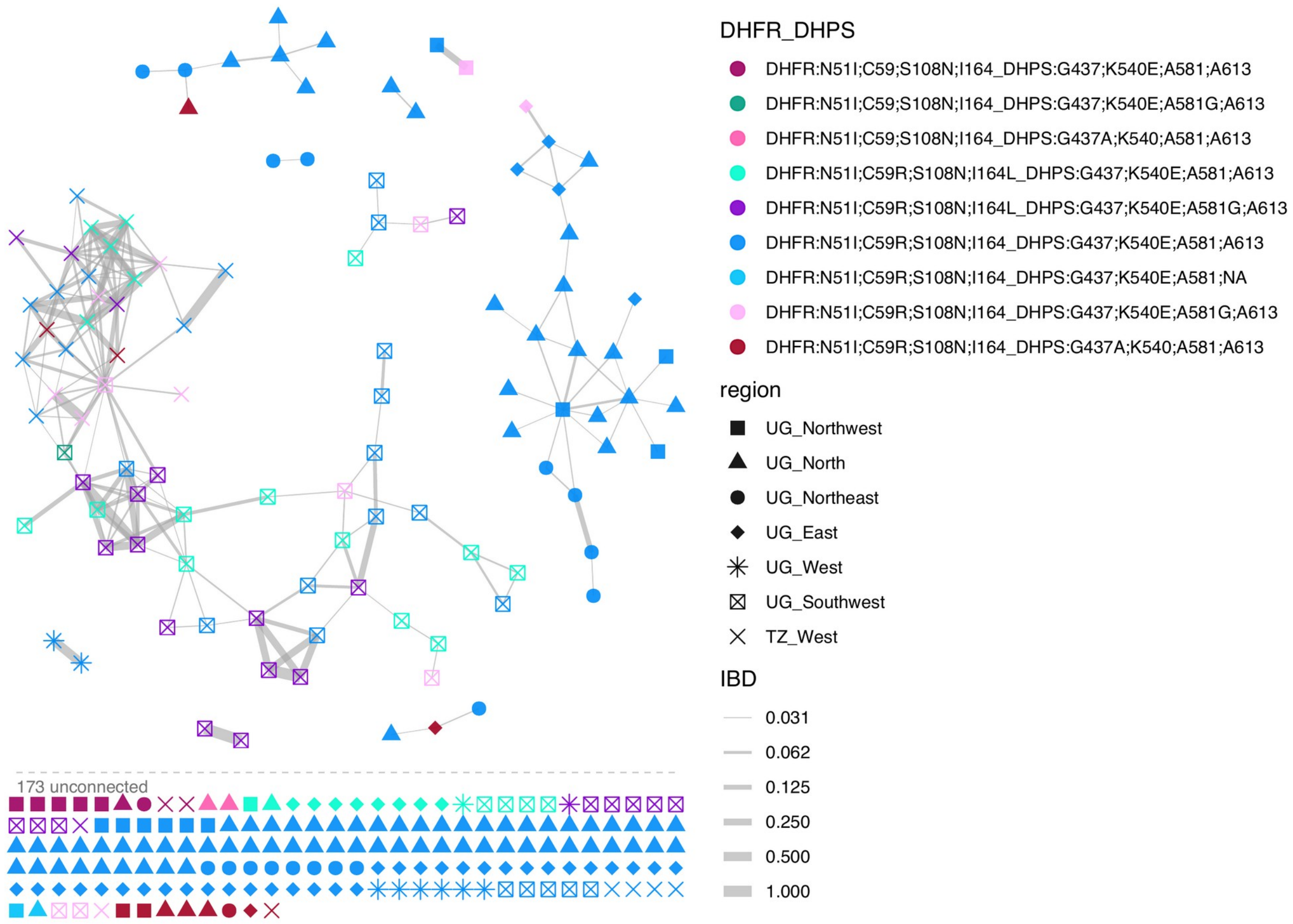

Supplementary Figure 5. Sample-level IBD network coloured by PfDHPS/PFDHFR and region. The nodes are coloured by the combined PfDHPS/PfDHFR sulfadoxine-pyrimethamine resistance haplotype. The network highlights the regional distribution of antifolate triple- and quintuple-mutant haplotypes and their sharing within IBD-connected clusters.

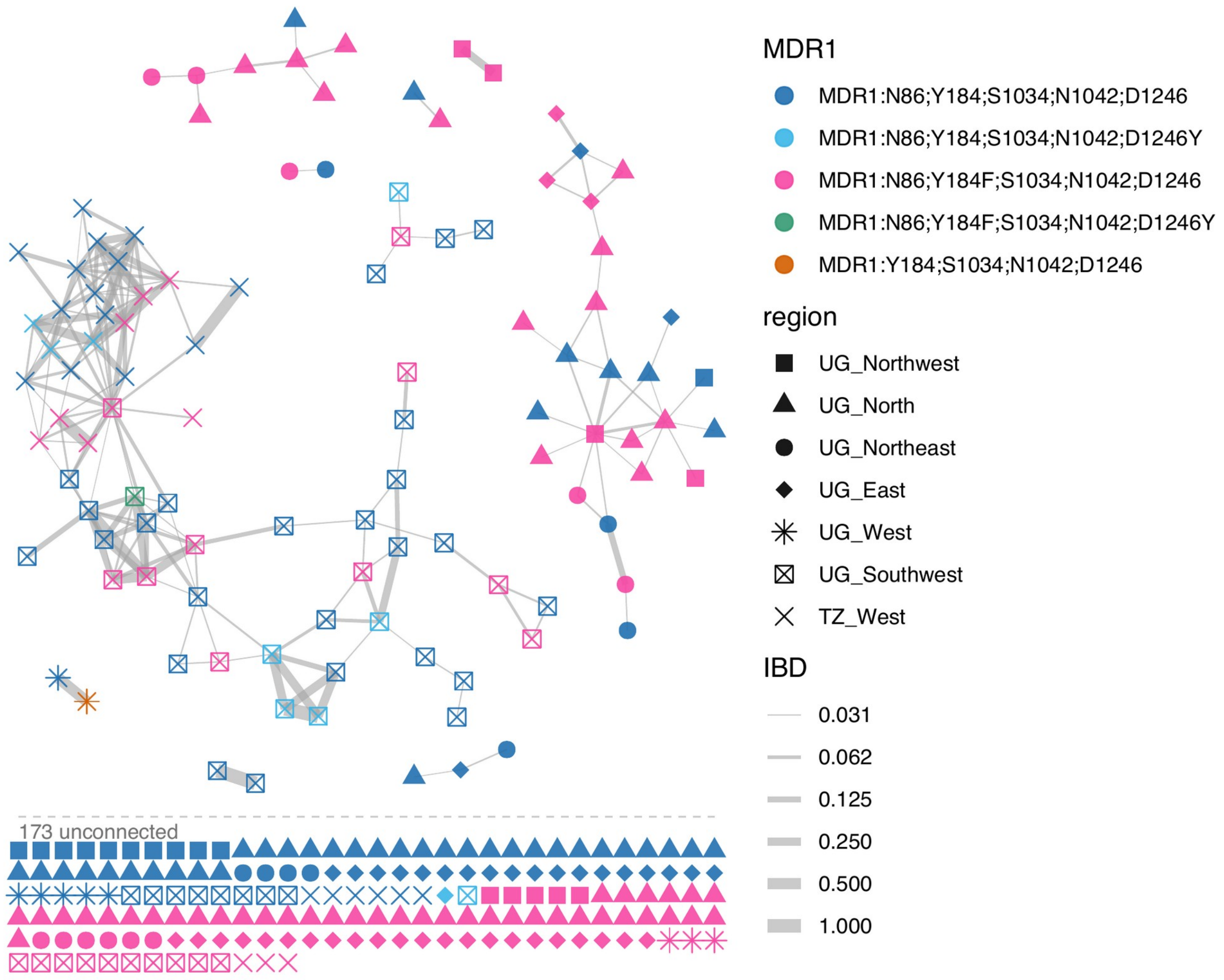

Supplementary Figure 6. Sample-level IBD network coloured by PfMDR1 haplotype and region. The nodes are coloured by PfMDR1 haplotype (N86Y, Y184F, D1246Y variants). The network illustrates how lumefantrine- and mefloquine-resistance associated alleles are distributed among related parasite lineages across Uganda's regions.

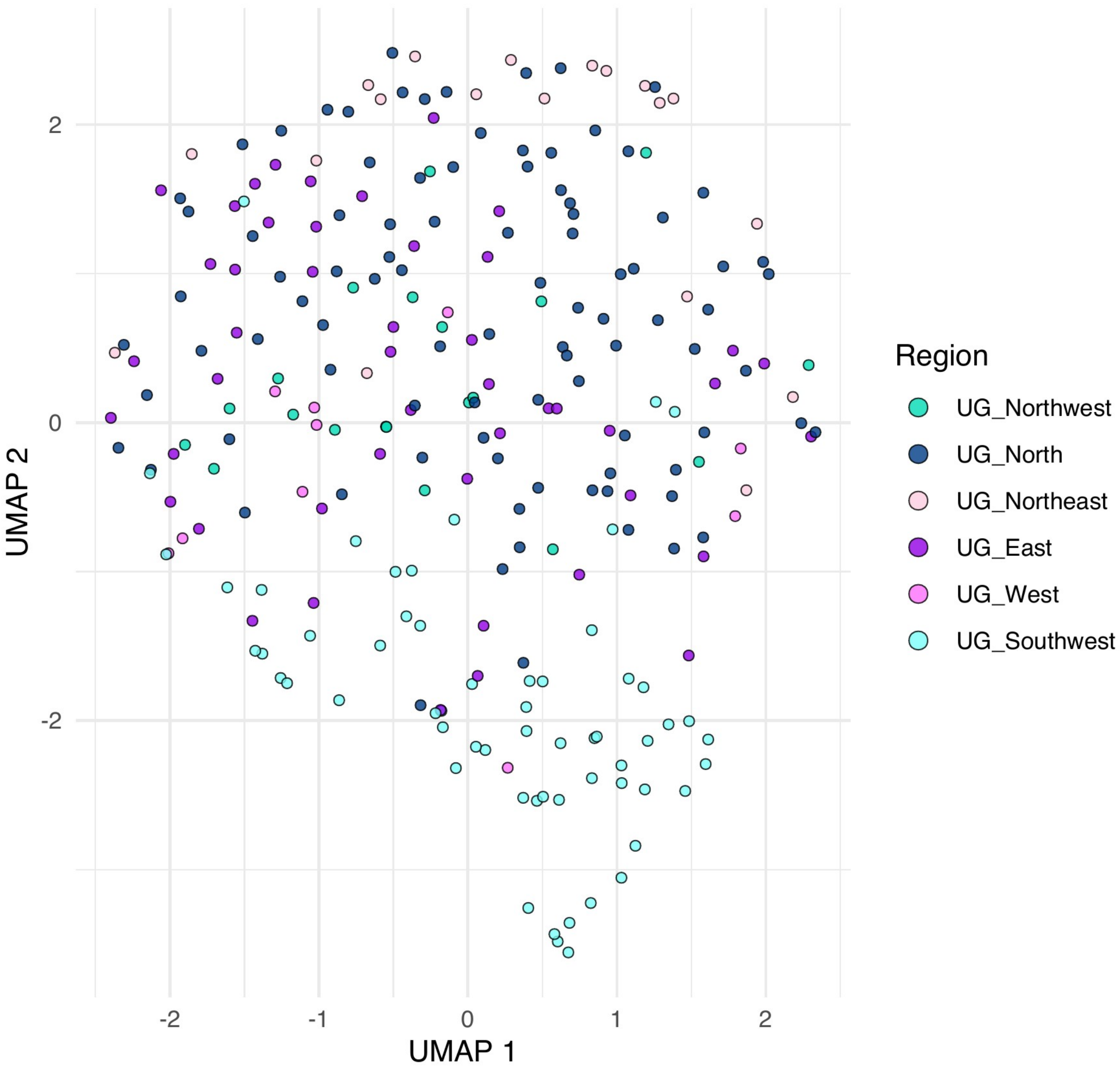

Supplementary Figure 7. UMAP projection of Uganda-only samples coloured by region. Uniform Manifold Approximation and Projection (UMAP) of genome-wide SNP variation restricted to the 249 Ugandan 2023 samples. Each point represents one sample, coloured by its region of collection. Clustering patterns reflect the degree of genetic differentiation among regions and identify any within-country population substructure. The only visible structure appears to be the Southwest samples which majority cluster together at the bottom of the larger cluster.

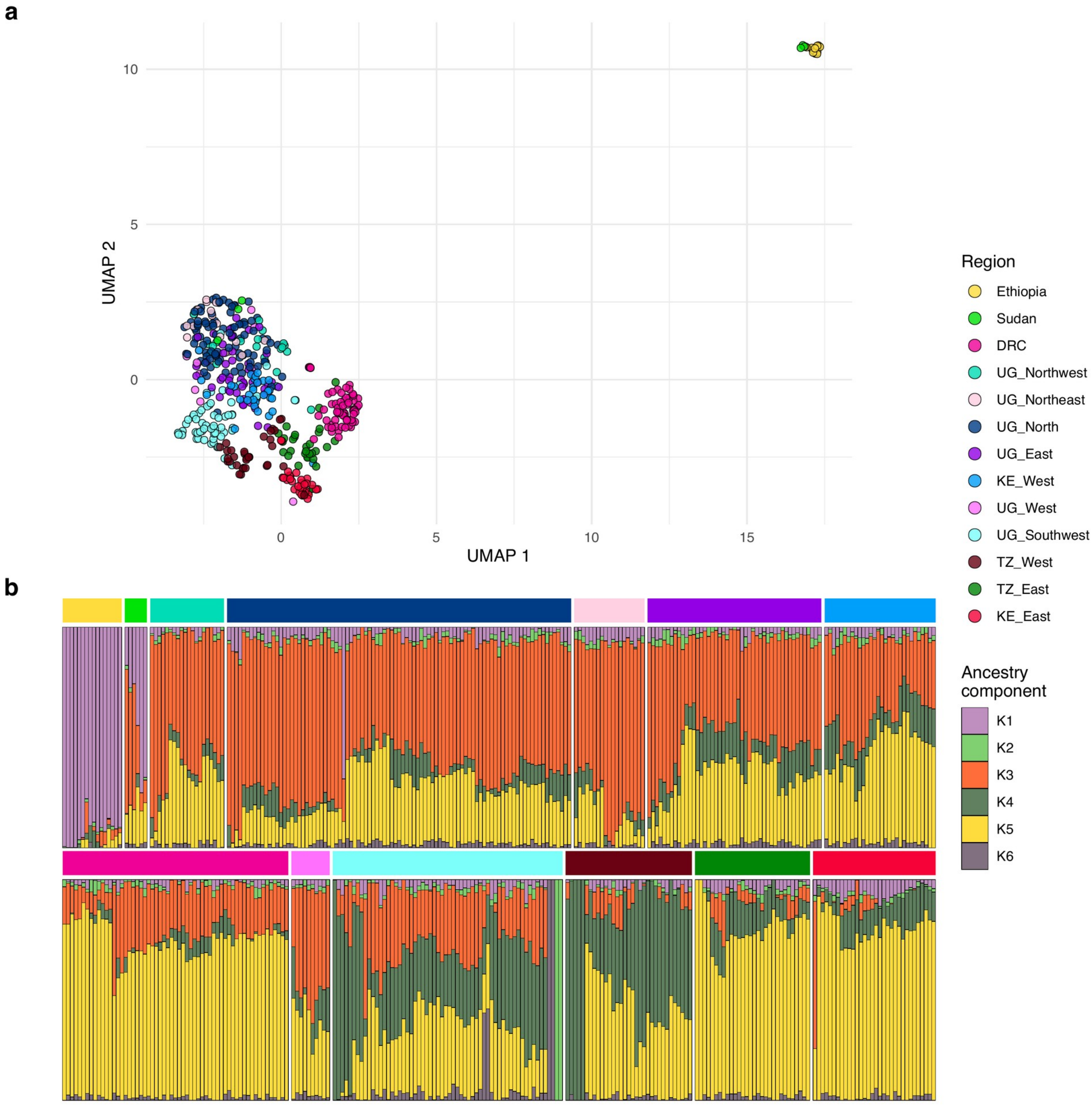

Supplementary Figure 8. Population structure of *Plasmodium falciparum* samples from Uganda and regional comparator populations. (A) UMAP of the samples from Uganda and comparator populations from Ethiopia, Sudan, Democratic Republic of the Congo (DRC), Kenya, and Tanzania coloured by region/population, showing genetic relationships and clustering patterns across Uganda and neighboring countries. **(B)** LEA sNMF admixture plot at K = 6 showing ancestry proportions for same samples. Each vertical bar represents one sample, and colours indicate inferred ancestry components. Three of the Sudanese parasites cluster with Uganda and one Ugandan parasite cluster with the Ethiopia/Sudan cluster, this parasite also is dominated by the K1 ancestry seen in the admixture for those countries, consistent with either with limited transmission in those regions with Northern Uganda or imported cases from travelers or refugees.

a

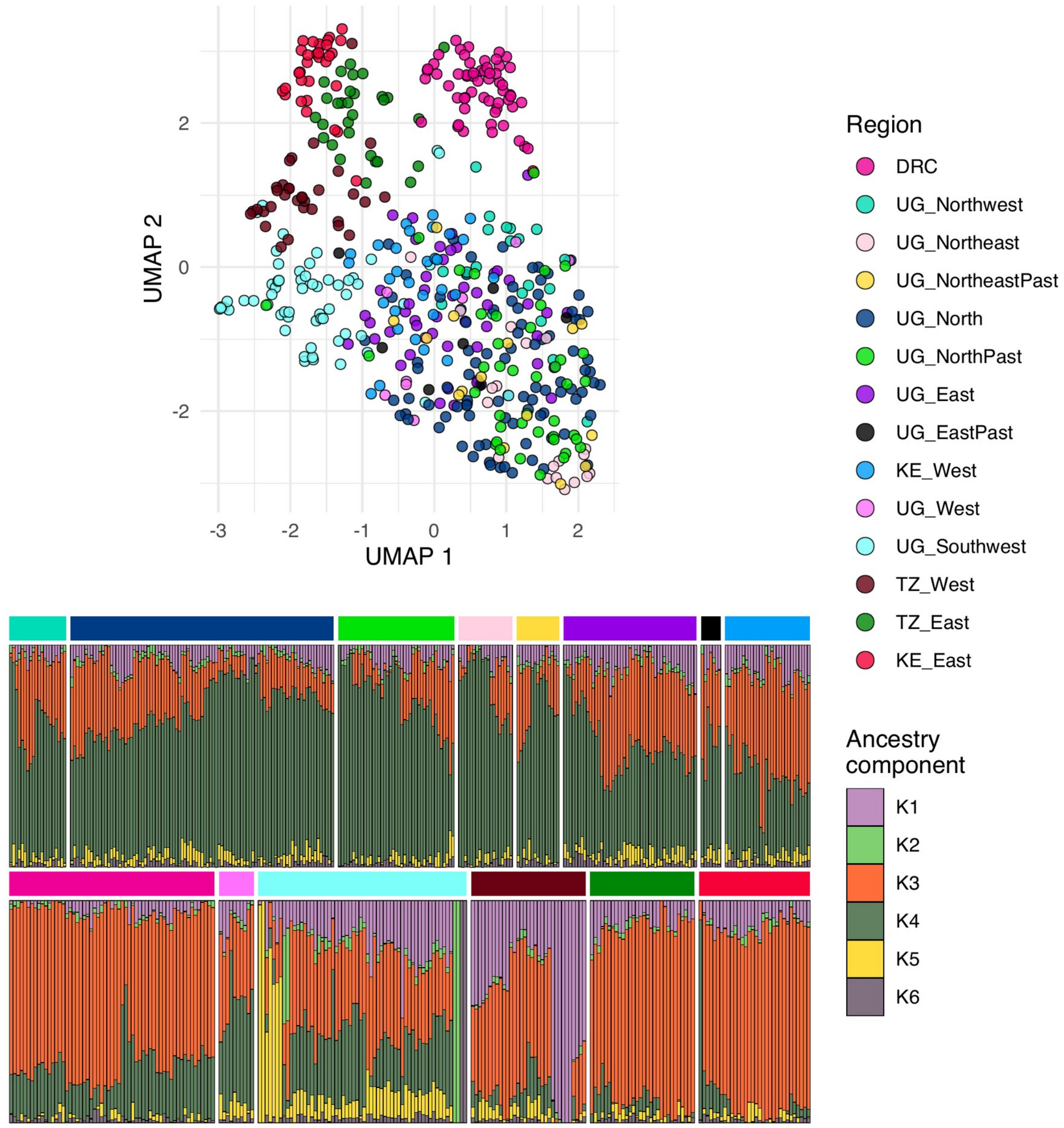

b

Supplementary Figure 9. Population structure of contemporary and past *Plasmodium falciparum* samples from Uganda in the context of regional comparator populations. a) UMAP of the samples coloured by region/group, illustrating genetic relationships between contemporary and past Ugandan parasites and their clustering relative to comparator populations from Democratic Republic of the Congo (DRC), Tanzania, and Kenya. Past data are only available for regions of Northeast, North and East and each of the past parasites have very similar ancestry components with their contemporary populations consistent with the parasite population not drastically changing over time. **b)** LEA sNMF admixture plot at K = 6 showing ancestry proportions for individual contemporary Ugandan samples, past Ugandan samples from selected regions, and comparator populations. Each vertical bar represents one sample, and colours indicate inferred ancestry components.

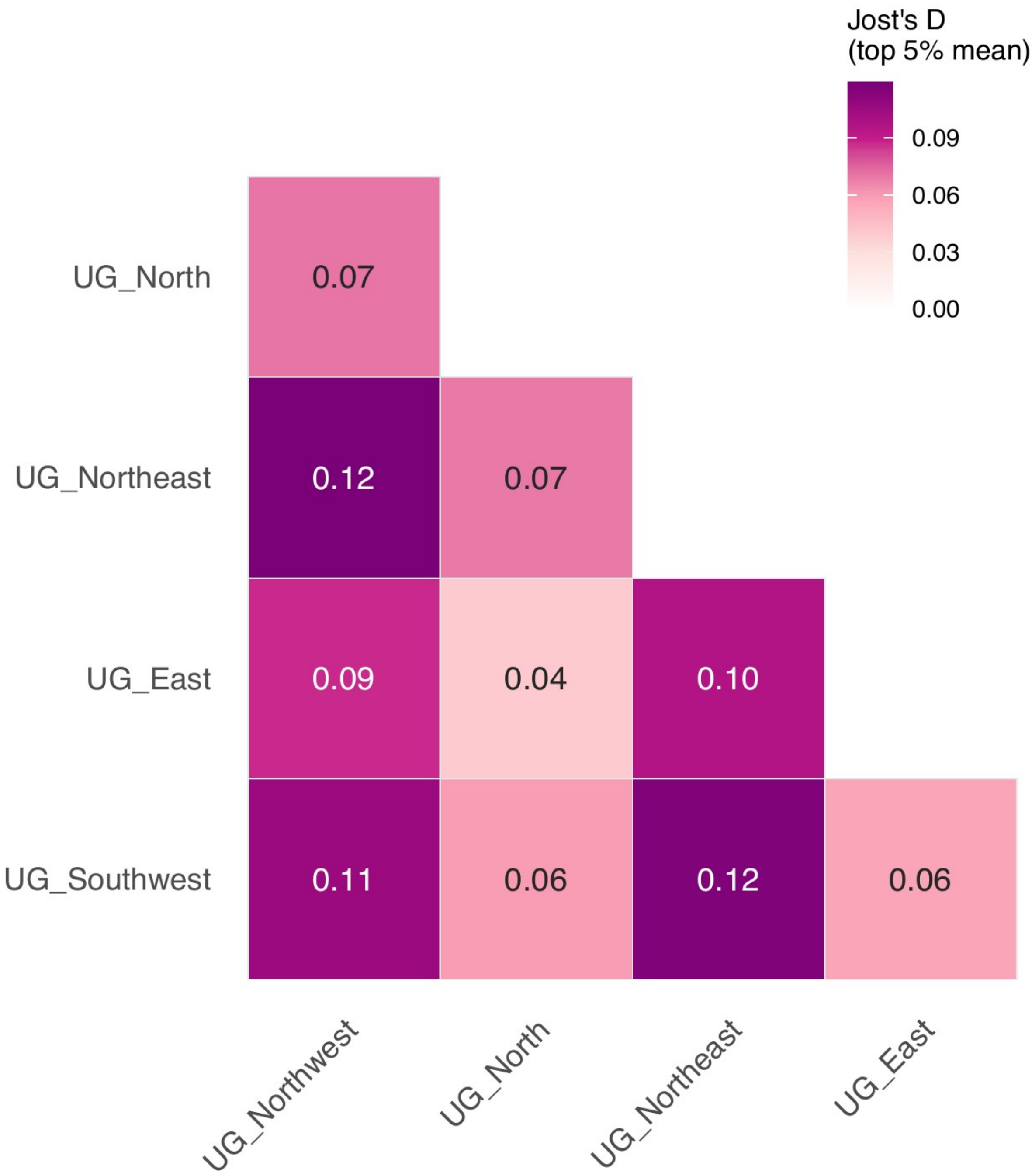

Supplementary Figure 10. Pairwise Jost's D heatmap by region. Colour-coded matrix of the mean pairwise top 5% of Jost's D differentiation statistics calculated between all region pairs. Higher values (darker colour) indicate greater allelic differentiation, providing a complementary measure of population structure to IBD-based analyses.

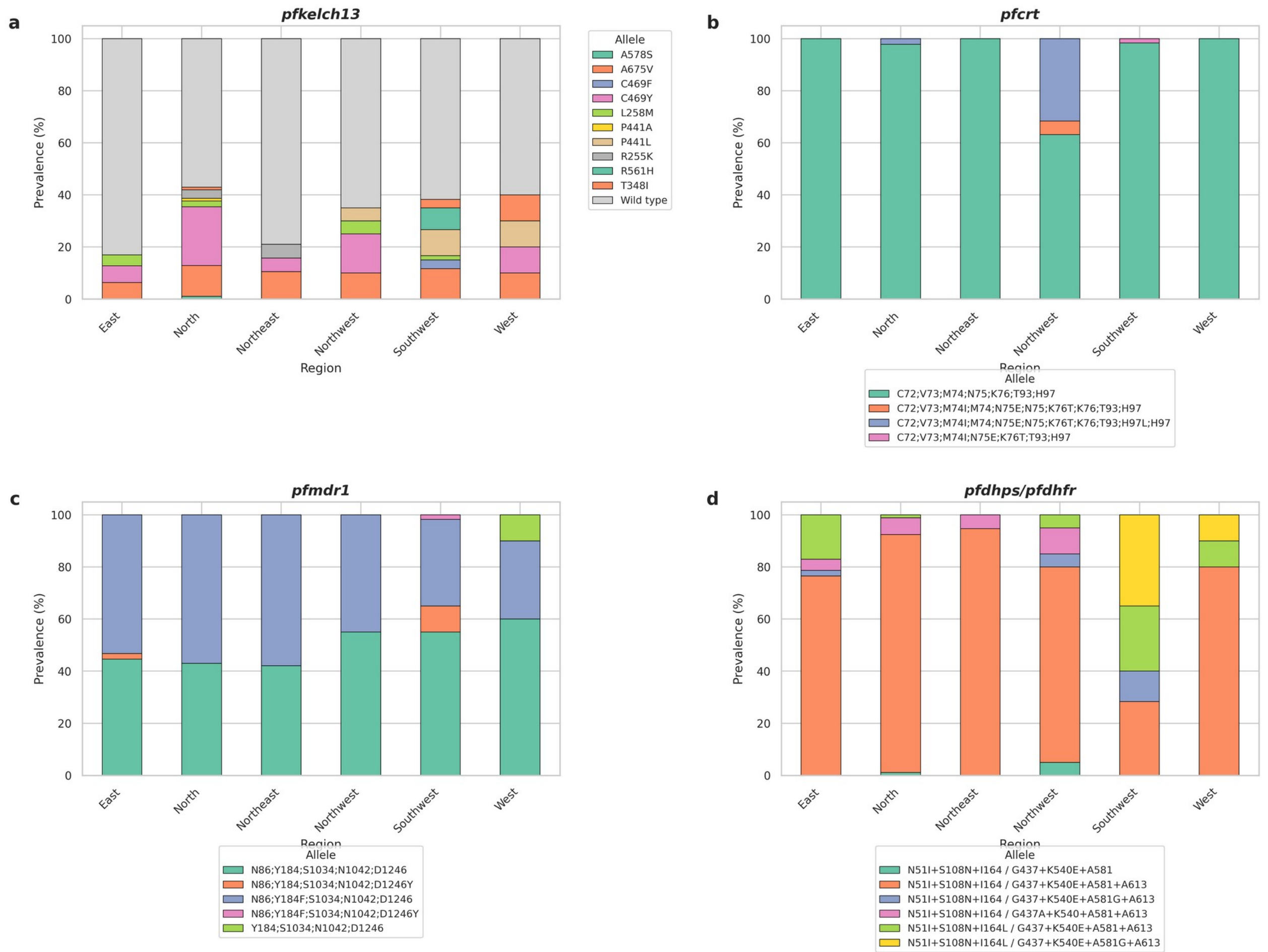

Supplementary Figure 11. Prevalence of drug resistance markers across PfKelch13, PfCRT, PfMDR1, and PfDHFR/PfDHPS by region. Bar charts summarizing the frequency of key antimalarial resistance mutations in each region. Markers include artemisinin partial-resistance candidates in PfKelch13, chloroquine-resistance alleles in PfCRT, multidrug-resistance alleles in PfMDR1, and antifolate-resistance haplotypes in PfDHFR and PfDHPS.

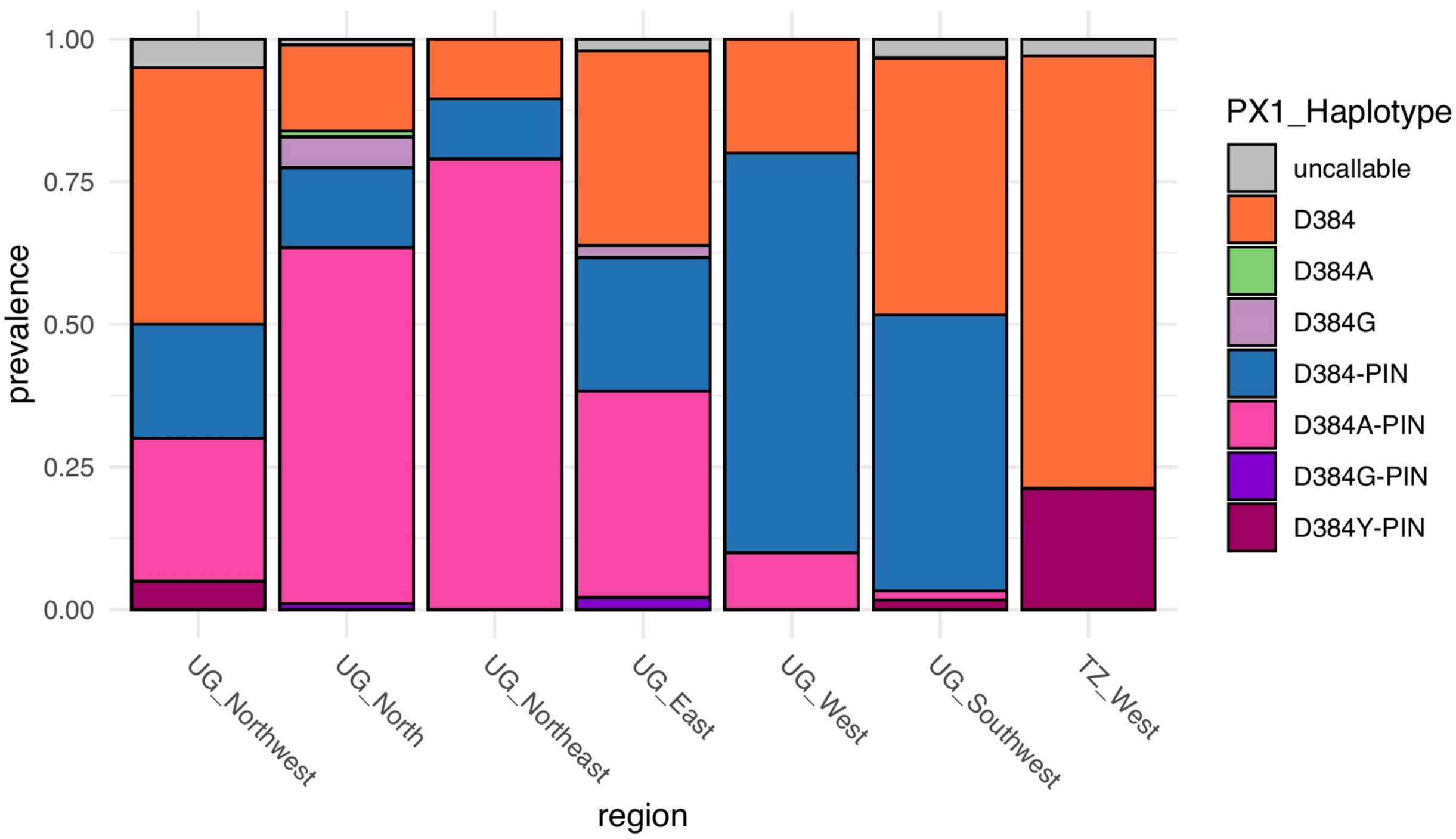

Supplementary Figure 12. PfPX1 Haplotype Prevalence. The prevalence of the PfPX1 D384A/G/Y, L1222P, M1701I, and D1705N haplotype across each region. The haplotypes are sorted by the presence or absence PIN. The total prevalence the PIN haplotype is the equivalent of they-axis height of the blue bar for the Ugandan samples and the dark maroon bar for the Tanzania West (TZ_West) samples.

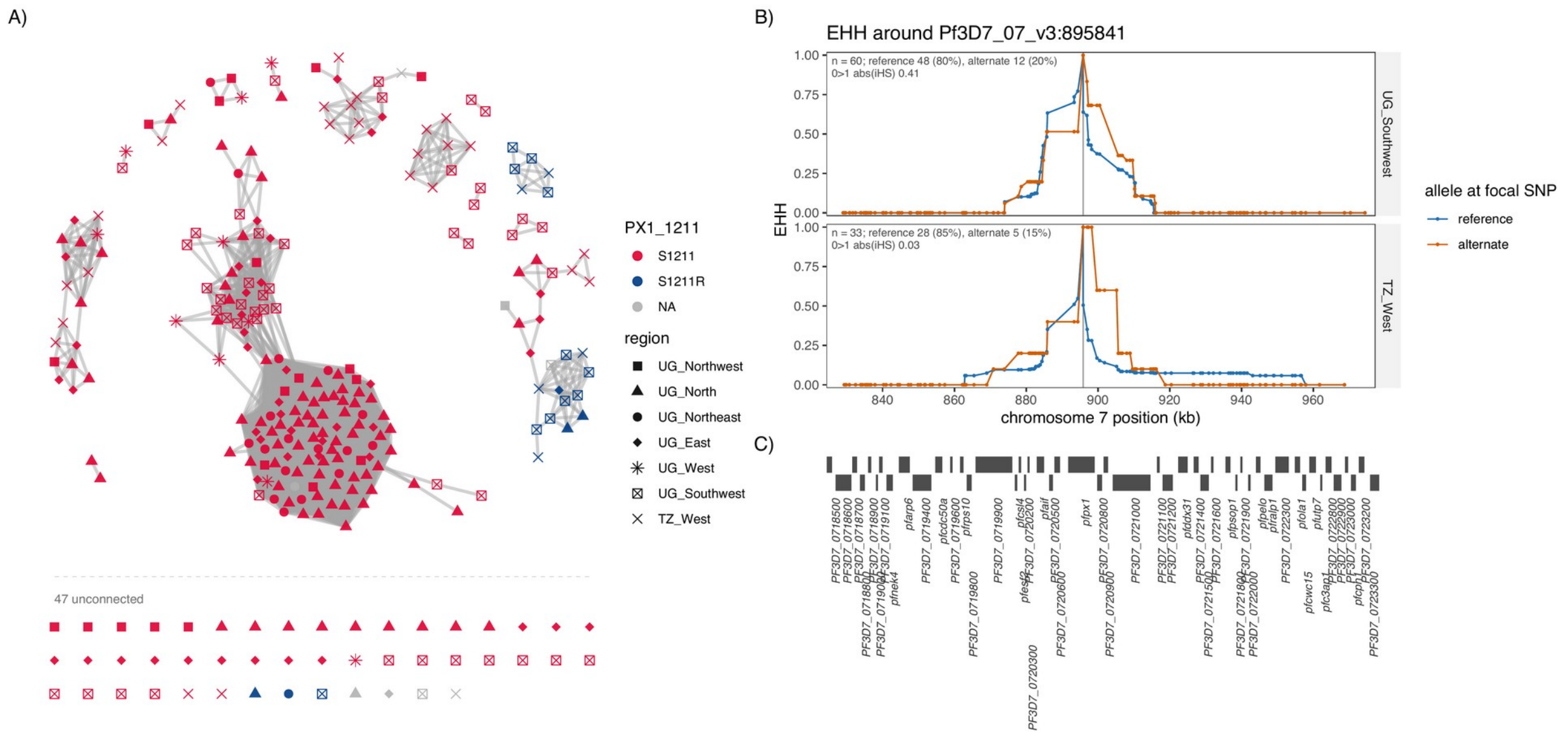

Supplementary Figure 13. PfPX1 S1211R mutation network and EHH. (A) The network between Uganda and Tanzania West samples. Connections are shown if samples have an IBD block that completely covers the PfPX 1211 corresponding genomic locus, colored by whether or not they contain S1211R and the shapes of the nodes are the region they are from. The S1211R mutation is found only in non-PIN *pfpx1* isolates. Almost all the S1211R samples have an IBD block with another sample. (**B)** Is the EHH for Uganda Southwest and Tanzania West. There is not a large extended haplotype around this mutant which would be consistent with the mutation not being selected for and

therefore not sweeping but instead simply drifting. This variant was uncommon among African isolates (3.0% prevalence in Burkina Faso, 2.7% in Nigeria, 1.5% Tanzania, and <1% elsewhere) in the MalariaGEN Pf8 dataset but reached 33% prevalence in Indonesia and 12% in Papua New Guinea.

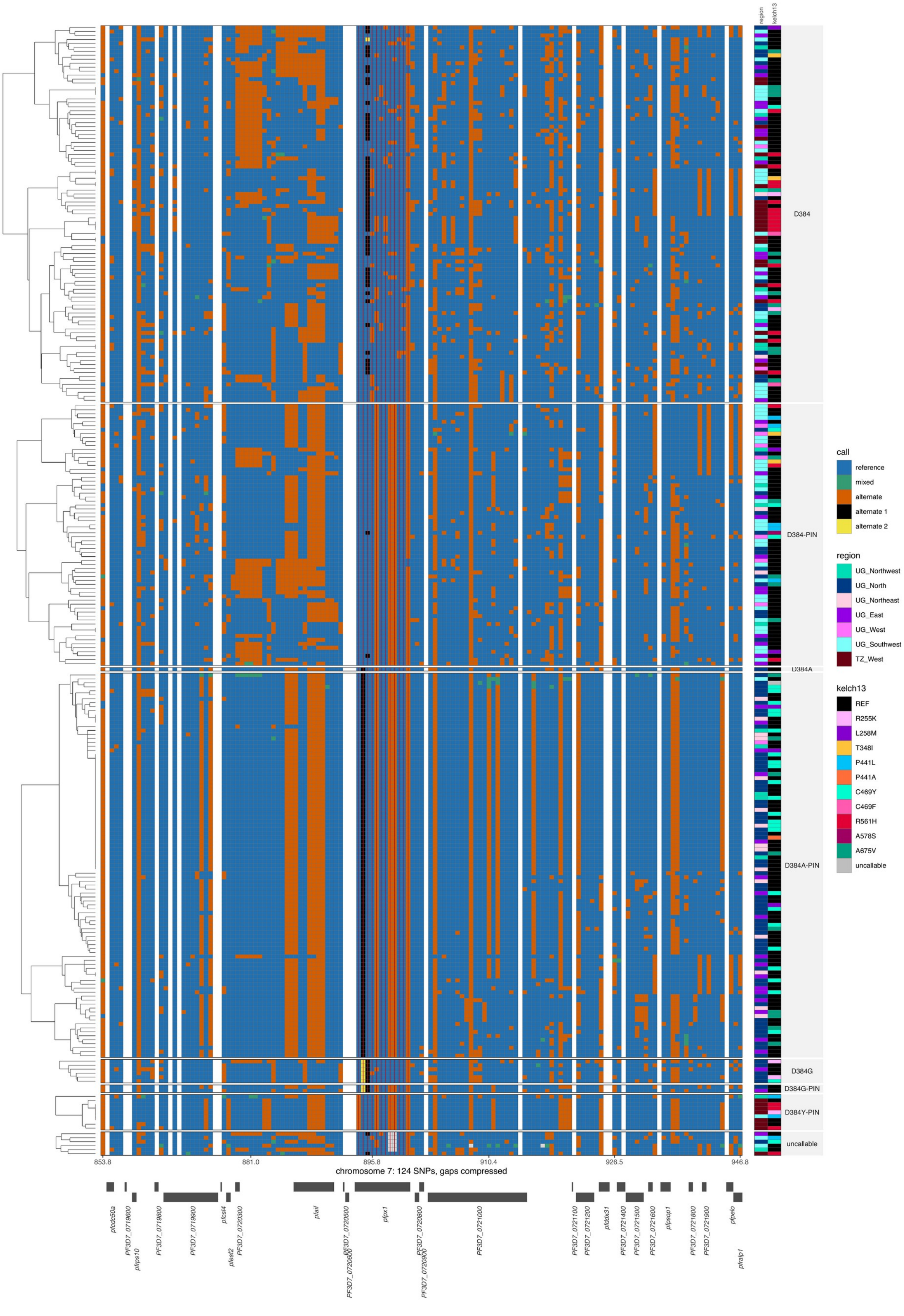

Supplementary Figure 14. Local genotypes around *pfpx1*. Each column is a called polymorphic single nucleotide position centered around *pfpx1,* extending left and right ~50kb. Each row is a sample clustered based on the variant calls and split by PfPX1 haplotype. Columns to the right annotate the regions the isolates come from and what PfKelch13 mutations they carry. D384A-PIN demonstrates a wider shared haplotype than D384-PIN. D384A-PIN also carries the majority of the PfKelch13 C469Y mutations. Like D384A-PIN, D384Y-PIN has a wide haplotype, but is predominately found in the west of Tanzania. D384G is found with PIN for 2 out of the 8 total D384G isolates and was only detected in the North and East regions.

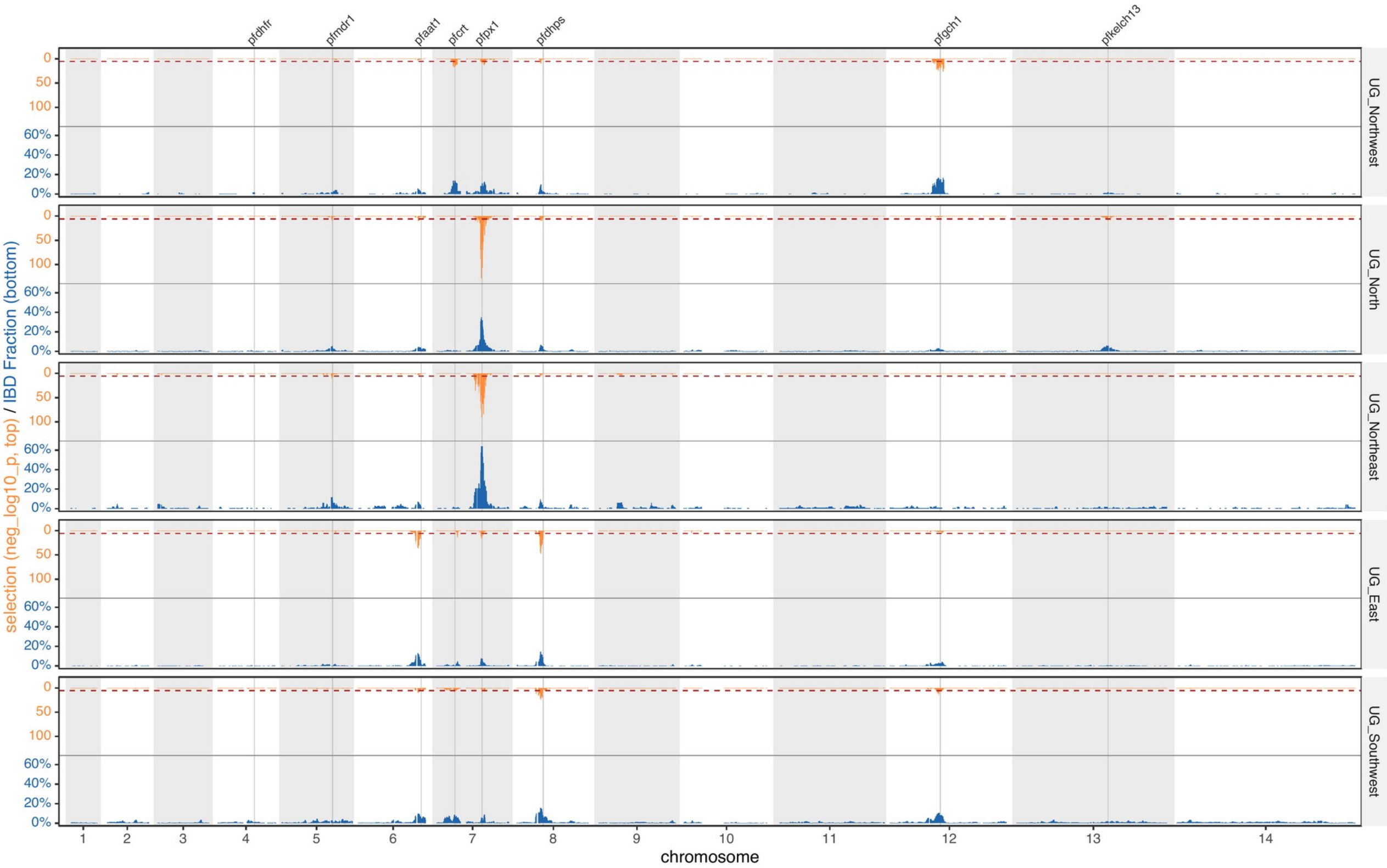

Supplementary Figure 15. Genome-wide IBD-based selection/pairwise sharing scans IBD by region. Above the central horizontal axis (orange), the IBD based selection statistic XiR,s SNPs with the red line marking where above SNPs were significant based on Bonferroni. Below the central horizontal axis (blue), the proportion of isolate pairs sharing IBD at each SNP position within each region. Candidate selection peaks corresponding to known drug resistance loci are labeled.

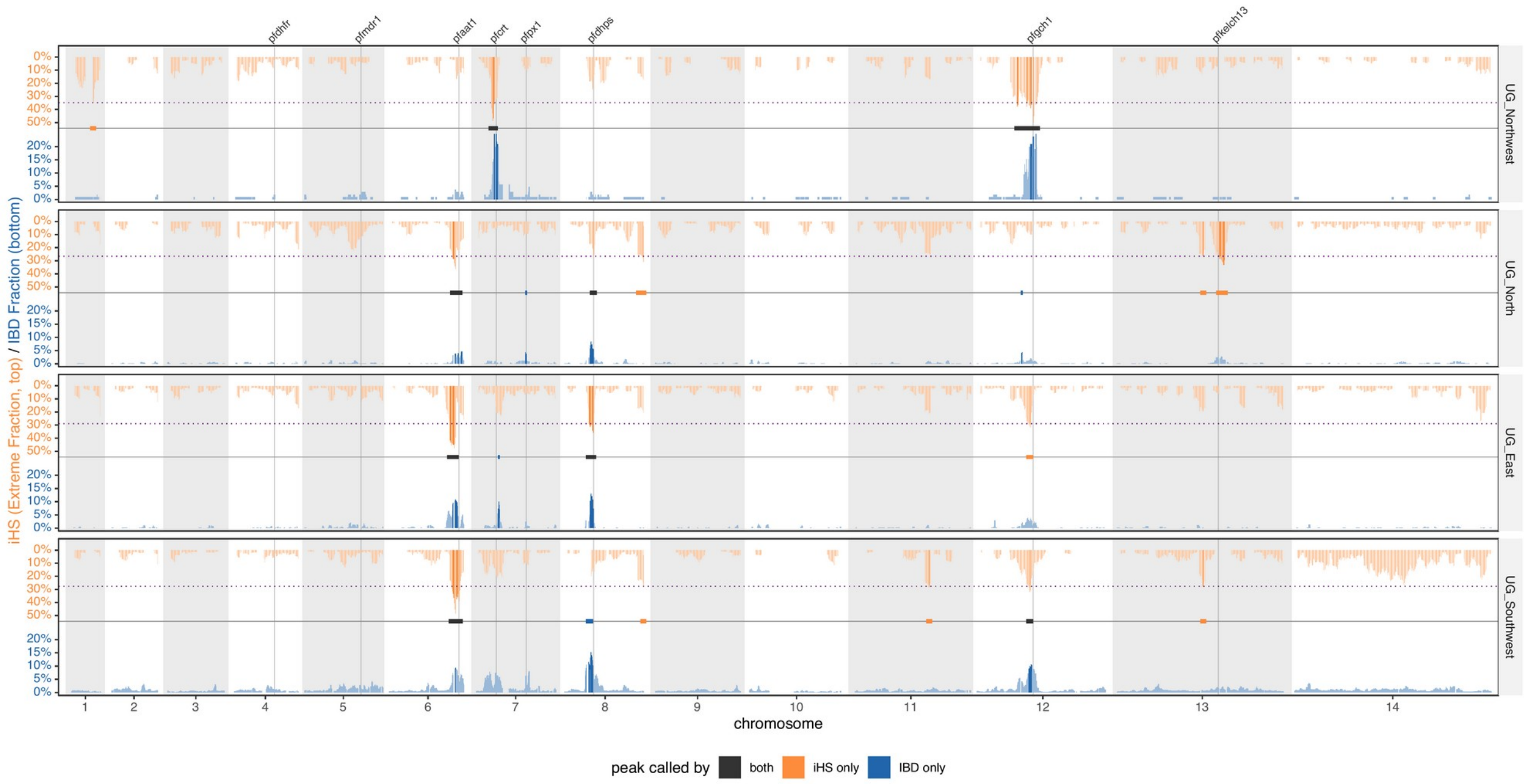

Supplementary Figure 16. Haplotype-based selection with Integrated Haplotype Score (iHS) mirrored against IBD sharing, by region all chromosomes. One panel per region. Above the axis (orange), the fraction of SNPs with |iHS| > 2 in 100-kb windows stepped every 10 kb; below (blue), the fraction of sample pairs sharing IBD at each SNP. Per-SNP iHS is dominated by sampling variance whereas a sweep elevates a contiguous run of SNPs, so selection is summarised as the proportion of extreme SNPs per window rather than SNP by SNP (Voight et al. 2006). iHS was computed in R package rehh, unpolarized so only |iHS| is interpreted, and standardised within 10 quantile bands of minor-allele frequency. Drug-resistance loci are marked. A purple dotted line marks the height of the top 1% genome wide of iHS peaks. The dominant signal is at *pfpx1* (chromosome 7), extreme in both tracks (iHS 67%, IBD 34% in UG_North; IBD 62% in UG_Northeast). Two further loci are elevated in both tracks in UG_Northwest: *pfgch1* (chromosome 12; iHS 42%, IBD 15%) and *pfcrt* (chromosome 7; iHS 35%, IBD 14%), the latter also moderately in UG_Southwest (iHS 20%, IBD 9%). Three loci are elevated in iHS with little corresponding IBD sharing: pfkelch13 (chromosome 13, 44% in UG_North), *pfaat1* (chromosome 6, 43% in UG_Southwest) and *pfdhps* (chromosome 8, 43% in UG_East). *pfdhfr* shows no signal in either track.

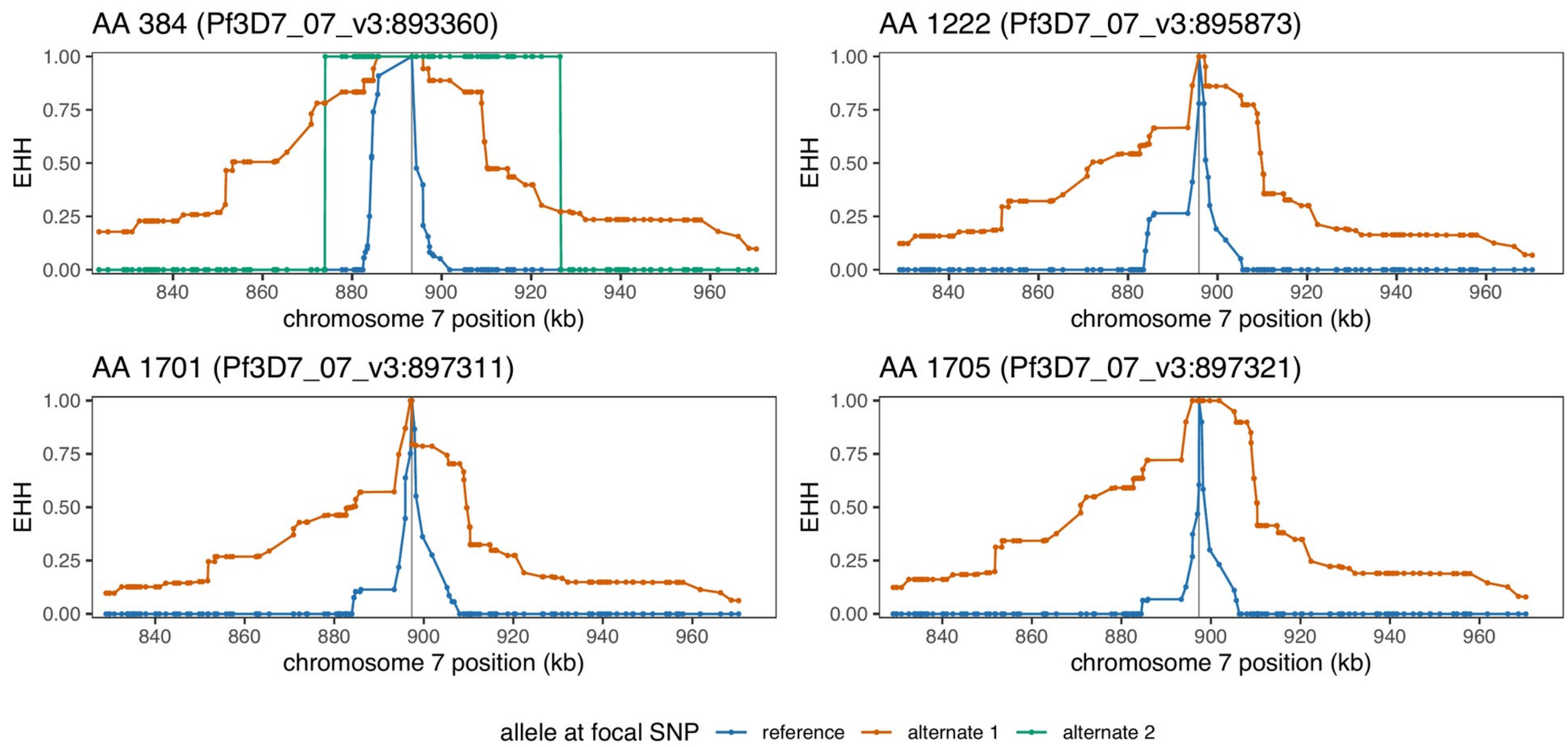

Supplementary Figure 17. EHH plots of PfPX1 variants in 2020-2021 Ugandan Isolates. Extended haplotype plots for Uganda 2020-2021 isolates of the 4 important residues seen in the sweeping D384A-PIN haplotype (384 top left, 1222 top right, 1701 bottom left, 1705 bottom right) created by wrapping R package rehh. Blue lines are the reference, and orange is the first alternative. Positions 1222, 1701, and 1705 are biallelic SNPS and only have 1 alternative, while 384 is a multiallelic SNP with a 2nd alternative allele (green line), for 384 the orange line is the allele coding for D384A, the green line is the alternative coding for D384G, the non-PIN associated 384 amino acid. The iHS score is based on the ratio of the area under the allele of interest to the reference allele. The longer the alternative allele's haplotype and/or the shorter the reference haplotype, the greater the iHS. The iHS scores from highest to lowest, D1705N 4.78 p=6.88e-06; D384A 3.84 p=4.92e-04; L1222P 3.72 p=7.91e-04; M1701I 3.55 p=0.00157; D384G 1.4 p=0.65.

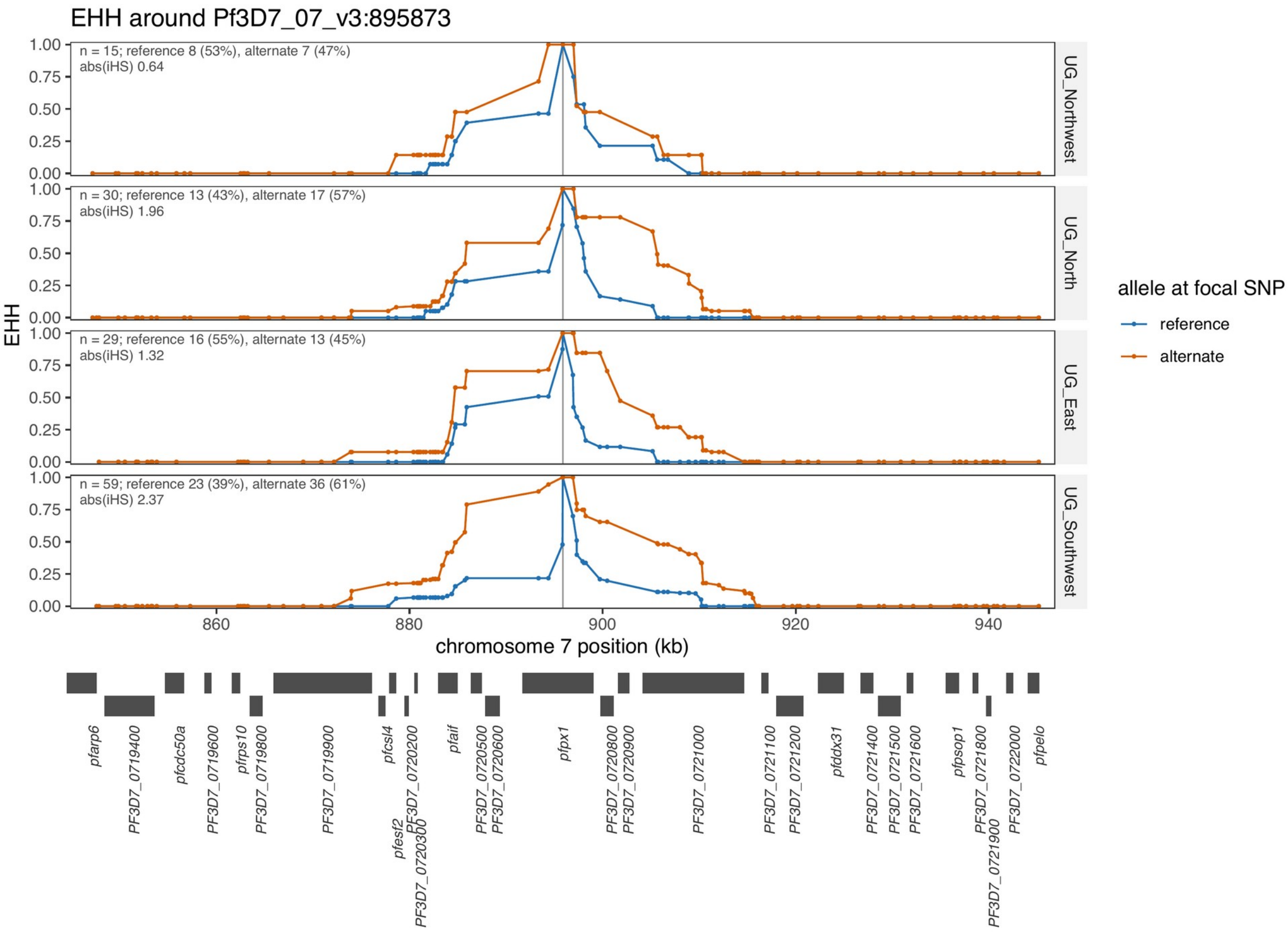

Supplementary Figure 18. EHH plots of PfPX1 variants with D384A-PIN haplotypes removed. After removing D384A-PIN haplotypes from the population and recalculating iHS and replotting EHH for 1222P, a very similar extended haplotype with similar mild iHS scores can be seen. There is also a similar prevalence across regions ranging from 45-60%. All of which would be consistent with a similar D384-PIN background from with D384A-PIN then arose.

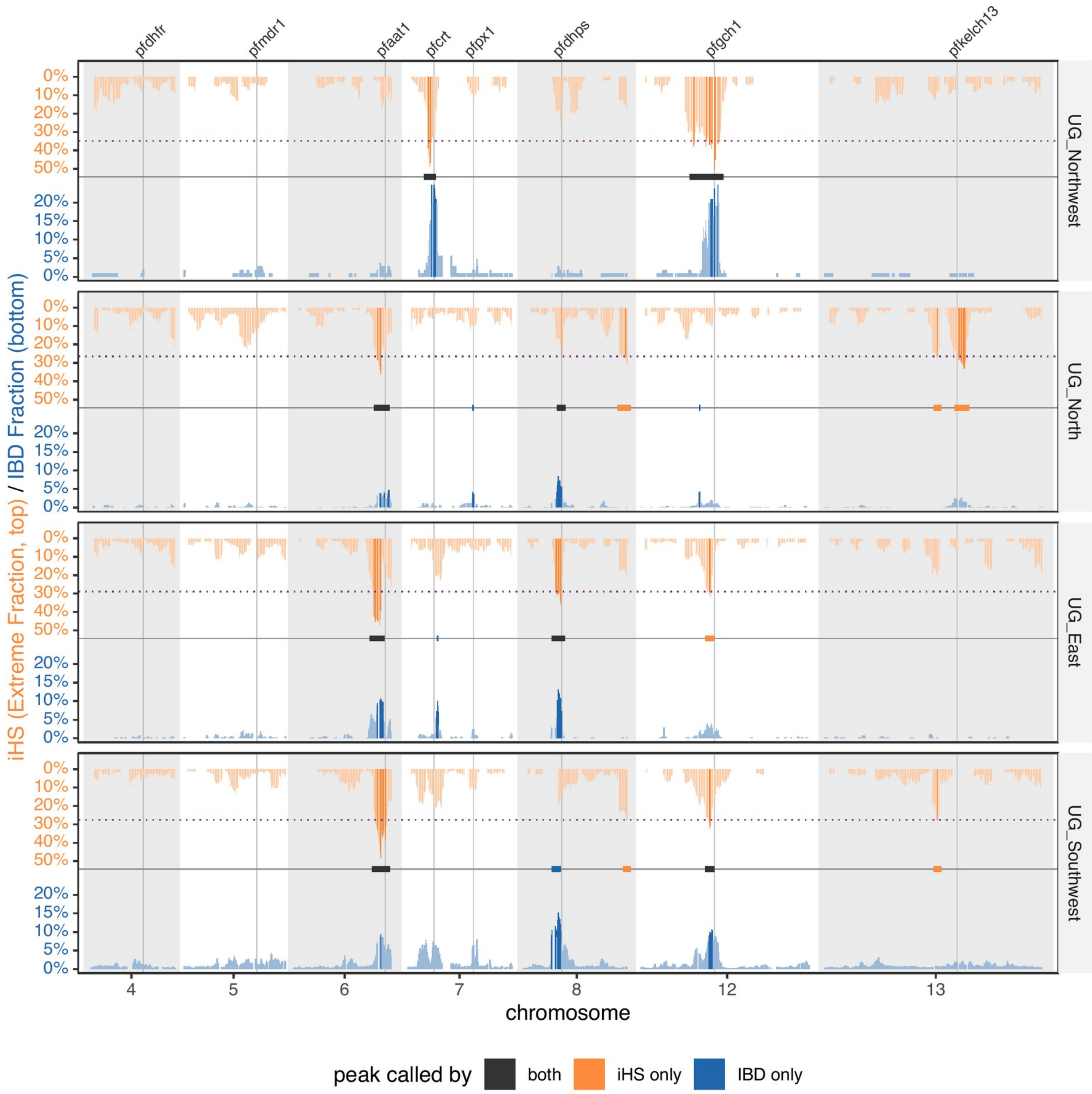

Supplementary Figure 19. Haplotype-based selection with Integrated Haplotype Score (iHS) mirrored against IBD sharing with D384A-PIN removed. After removing D384A-PIN from the population and recalculating IBD and iHS there is almost no significant signal detected at *pfpx1* loci despite a prevalence of 45-60% D384-PIN left in the remaining population.

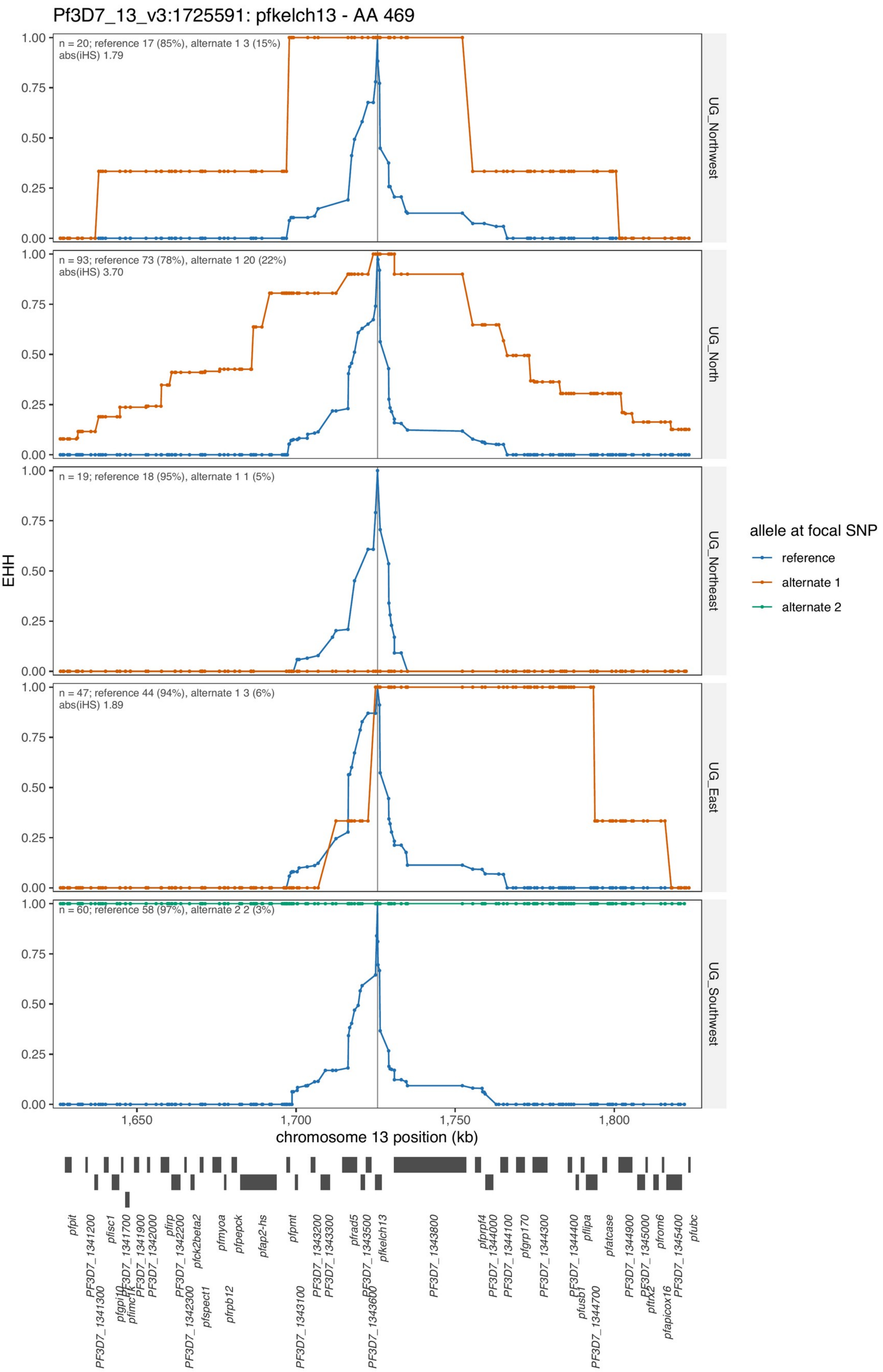

Supplementary Figure 20. EHH for PfKelch13 C469Y/F. Long haplotypes seen around C469Y (alternative 1) in the North (|iHS| 3.70) and Northwest (|iHS| 1.79). C469F (alternative 2) only seen in two isolates and share the same haplotype.

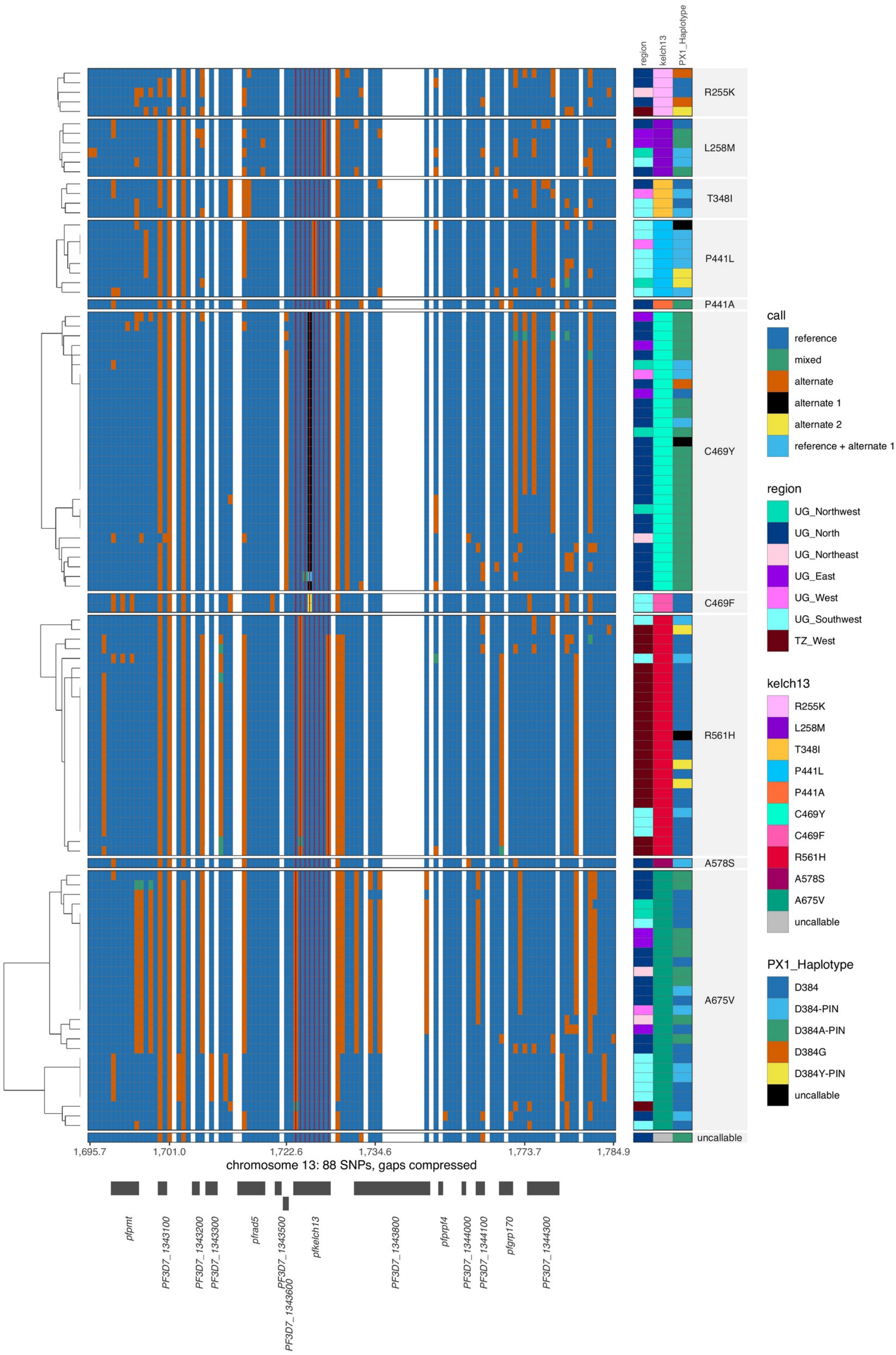

Supplementary Figure 21. *pfkelch13* genotypes. Each column is a variant SNP called position centered around *pfkelch13* extended left and right 100kb total. For every 2kb between SNPs that lack a SNP, a white column is placed to achieve relative genomic distance. Each row is a sample clustered based on the variant calls and split by their *pfkelch13* mutants (isolates containing reference left out). Distinctive haplotypes are found for several variants including A675V and R561H. R561H is found within Uganda Southwest and Tanzania West with at least two haplotypes.

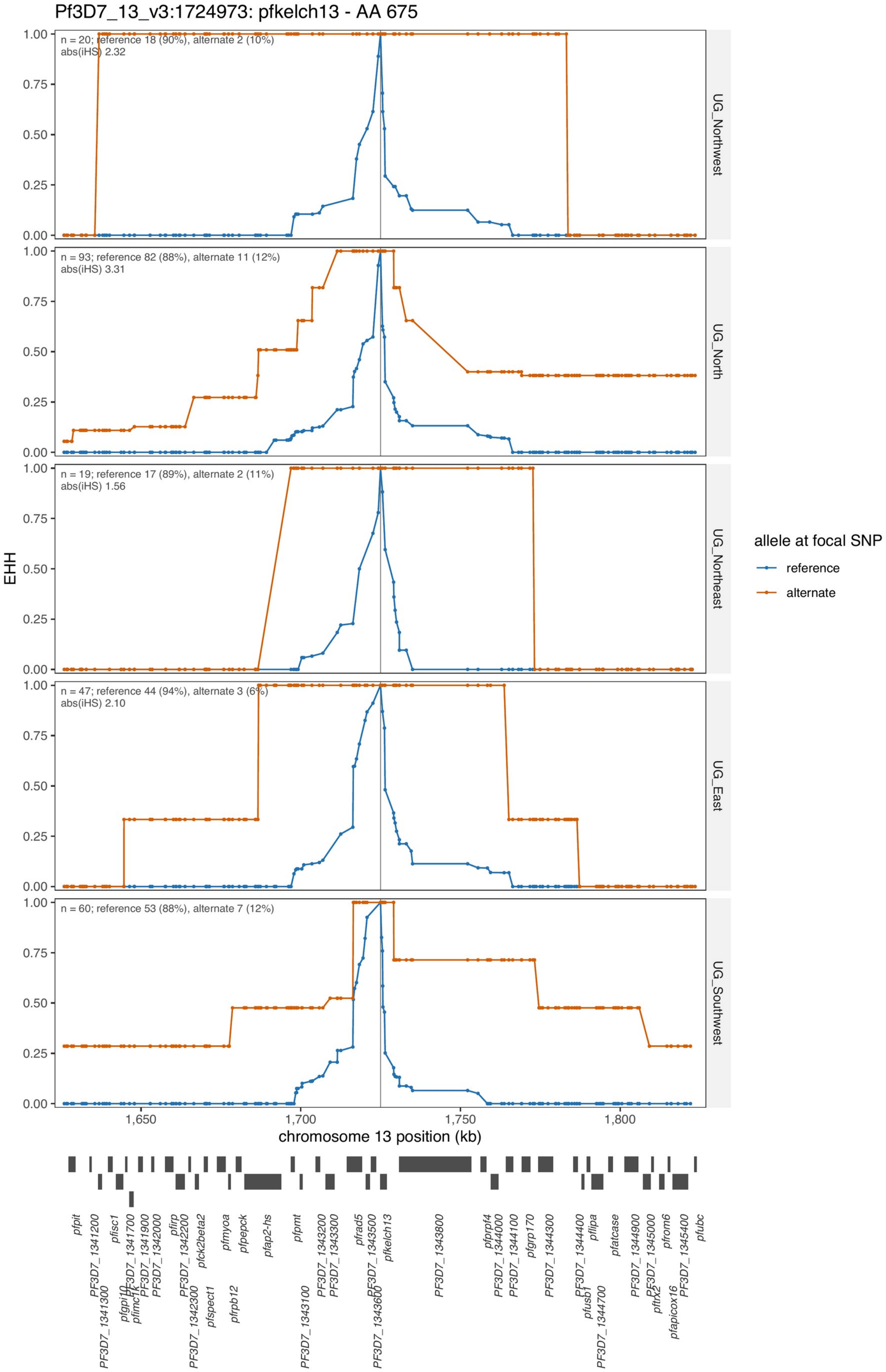

Supplementary Figure 22. EHH for PfKelch13 675. Long haplotypes seen around A675V (alternative 1) in the Northern/East region and Northwest as well for the haplotype in the Southwest.

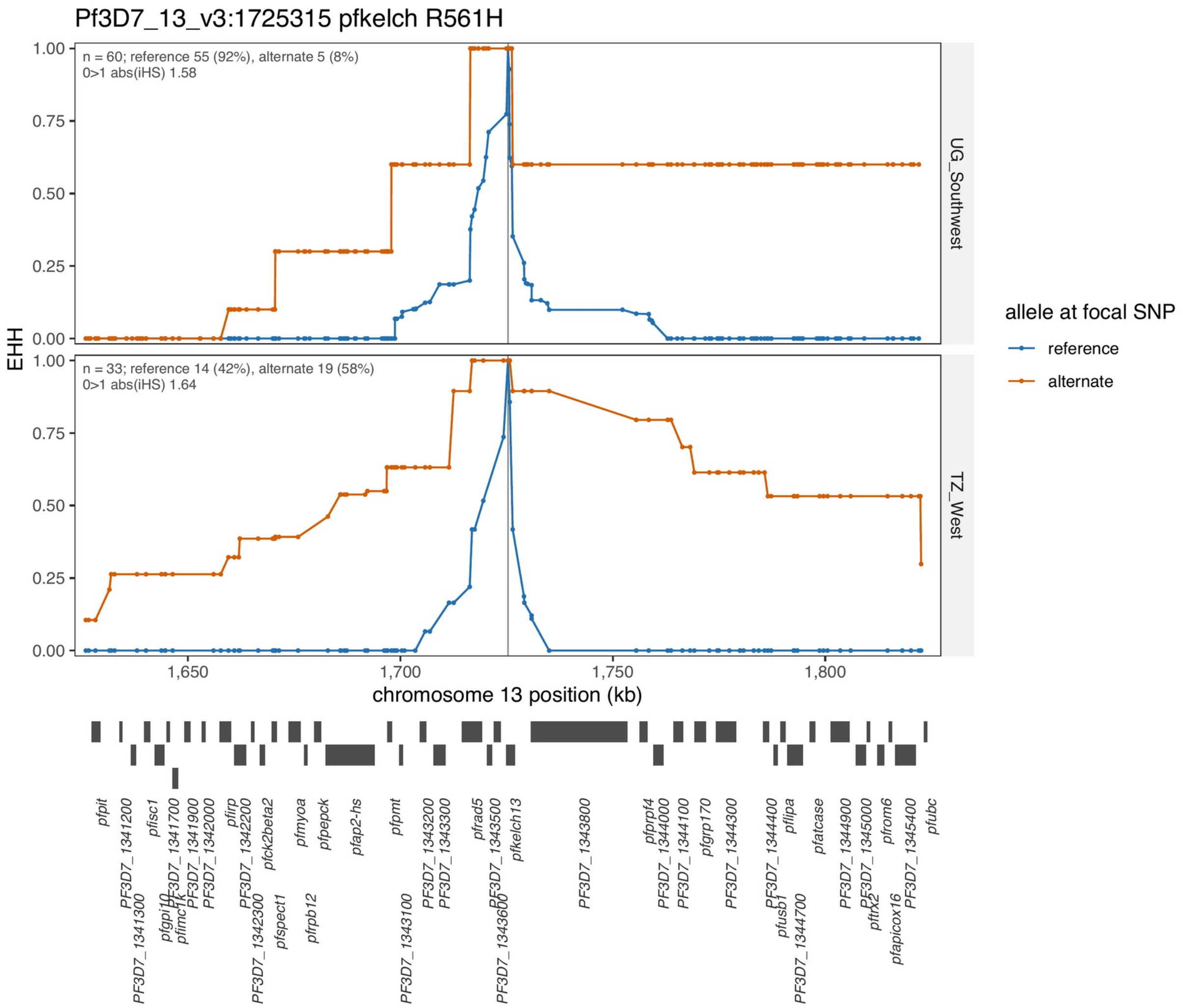

Supplementary Figure 23. EHH for PfKelch13 R561H. The EHH for the locus encoding for PfKelch13 R561H in Uganda Southwest and Tanzania West.

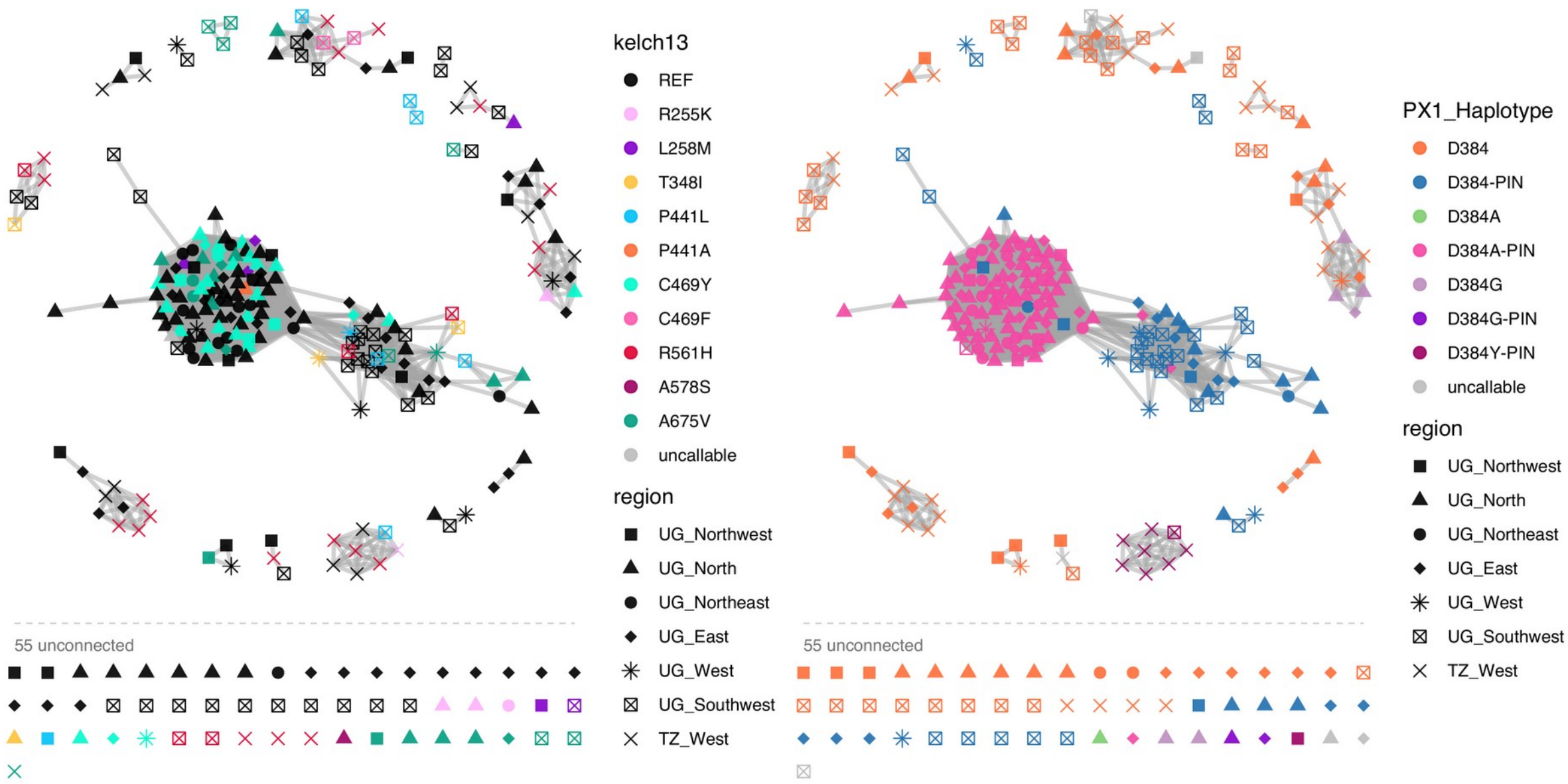

Supplementary Figure 24. PfPX1 networks colored by PfKelch13 mutations and by PX1 Haplotype. Association between the D384A-PIN vs D384-PIN vs everything else for PfKelch13 markers of artemisinin partial resistance (C469F, and A675V) was assessed countrywide by Fisher's exact test. Within the northern pool (North/Northwest/Northeast/East), where the D384A-PIN haplotype is essentially confined (81/83 isolates), C469Y was enriched on the D384A-PIN background relative to all other callable haplotypes (28.4% vs 6.3%; OR 5.8, 95% CI 1.8–24.4; BH-adjusted $p = 0.004$) and showed the same trend relative to D384-PIN specifically (28.4% vs 7.7%; OR 4.7, 95% CI 1.0–44.3; adjusted $p = 0.067$); A675V was unassociated with any PfPX1 variation. Although not statistically significant, 7 of the 8 P441L isolates identified countrywide carried the PIN haplotype; the small number of P441L observations limits the power of this comparison.

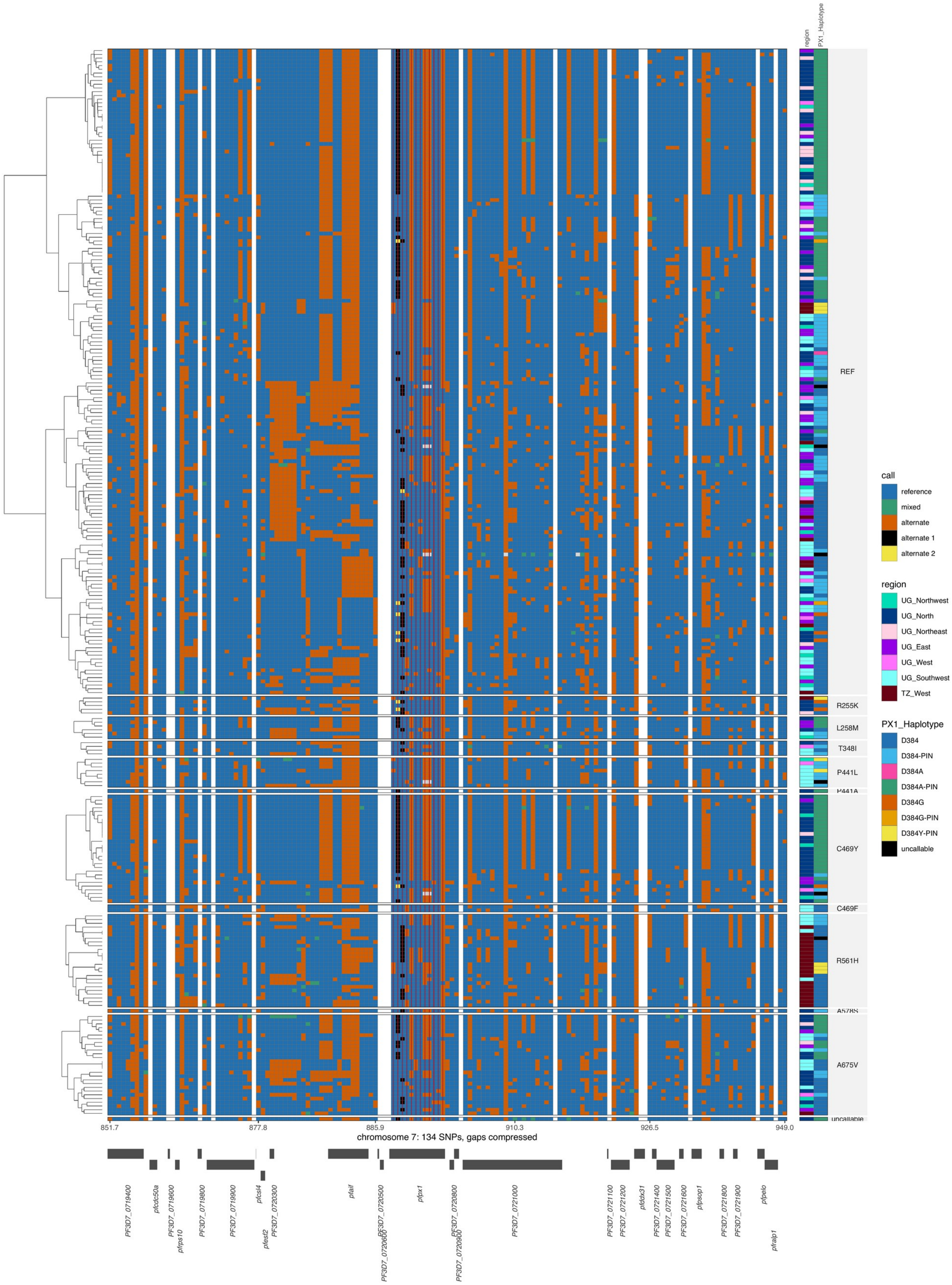

Supplementary Figure 25. *pfpx1* genotypes sorted by PfKelch13 mutations. The genotypes surrounding the *pfpx1* location split by PfKelch13 mutation. C469Y has almost all 384A-PIN haplotype. Seven out of 8 of the P441L have the PIN haplotype as well though the haplotypes for this mutation do not seem as wide as C469Y.

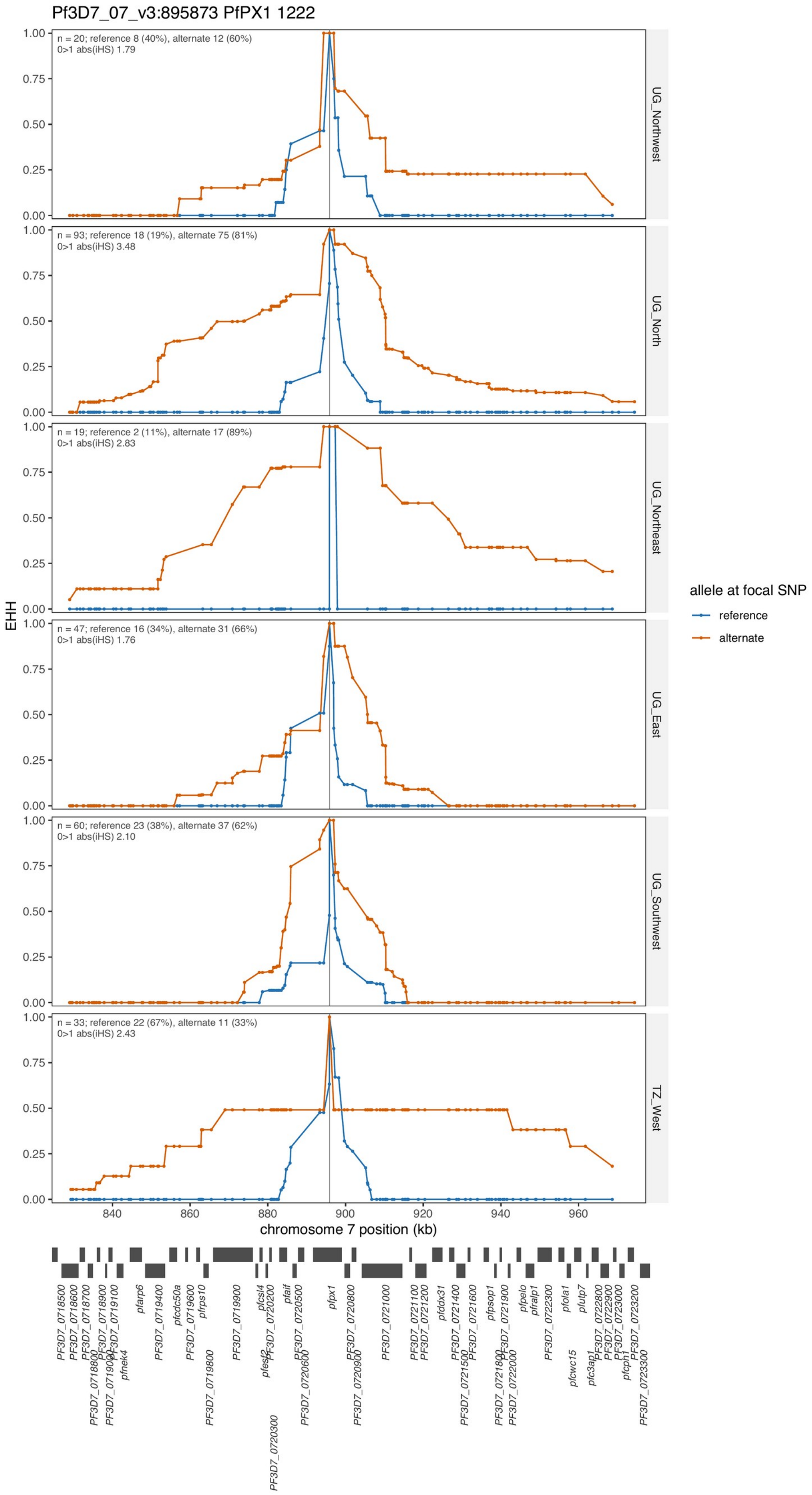

Supplementary Figure 26. Extended haplotype around *pfpx1* L1222P. Plotting the extended haplotype around the genomic locus that encodes for PfPX1 L1222P (variant P of the PIN haplotype). The haplotype is much wider in the North and Northeast where D384A-PIN is concentrated.

Supplementary Tables

Supplementary Table 1. Summary of genome-wide genetic diversity metrics (Nucleotide diversity, expected heterozygosity, and haplotype diversity) across six Ugandan regions.

Region	*n*	Nucleotide diversity ($\pi \times 10-4$)	Expected heterozygosity (*He*)	Haplotype diversity
East	47	2.34	0.149	0.966
North	93	2.36	0.152	0.991
Northeast	19	2.30	0.145	0.850
Northwest	20	2.30	0.143	0.854
Southwest	60	2.39	0.154	0.973
West	10	2.27	0.136	0.714

Nucleotide diversity (π): average number of pairwise nucleotide differences per site among sampled genomes; **Expected heterozygosity (*He*)**: probability that two randomly selected alleles at a locus are different; **Haplotype diversity**: probability that two randomly selected haplotypes are different.

Supplementary Table 2. Metadata for comparator and historical Plasmodium falciparum samples included in the study

Sample	District/Zone	Region	Country	African region	Collection year	Sequence accession
PZ0001-CW	Apac	Uganda_North-Past	Uganda	East Africa	2010	ERR063571
PZ0004-CW	Apac	Uganda_North-Past	Uganda	East Africa	2010	ERR063574
PZ0007-C	Apac	Uganda_North-Past	Uganda	East Africa	2010	ERR063577
PZ0008-CW	Apac	Uganda_North-Past	Uganda	East Africa	2010	ERR063578
PZ0010-CW	Apac	Uganda_North-Past	Uganda	East Africa	2010	ERR063580
PZ0015-C	Apac	Uganda_North-Past	Uganda	East Africa	2010	ERR216476
Pf003_HCMHWCCXY_L2	Mfangano	Kenya_West	Kenya	East Africa	2015	ERR6908269
Pf004_HFKVJCCXY_L6	Mfangano	Kenya_West	Kenya	East Africa	2015	ERR6908270
Pf005_HFKVJCCXY_L6	Mfangano	Kenya_West	Kenya	East Africa	2015	ERR6908271
Pf006_HCMHWCCXY_L2	Mfangano	Kenya_West	Kenya	East Africa	2015	ERR6908272
Pf007_HFKVJCCXY_L6	Mfangano	Kenya_West	Kenya	East Africa	2015	ERR6908273
Pf011_HCMHWCCXY_L2	Mfangano	Kenya_West	Kenya	East Africa	2015	ERR6908274
Pf012_HKCCLCCXY_L3	Suba	Kenya_West	Kenya	East Africa	2015	ERR6908275
Pf013_HK7MKCCXY_L3	Suba	Kenya_West	Kenya	East Africa	2015	ERR6908276
Pf015_HK7LVCCXY_L3	Suba	Kenya_West	Kenya	East Africa	2015	ERR6908277
Pf017_HKCCLCCXY_L3	Suba	Kenya_West	Kenya	East Africa	2015	ERR6908279
Pf018_HK7MKCCXY_L3	Suba	Kenya_West	Kenya	East Africa	2015	ERR6908280
Pf019_HK7MKCCXY_L3	Suba	Kenya_West	Kenya	East Africa	2015	ERR6908281
Pf020_HK7MKCCXY_L3	Suba	Kenya_West	Kenya	East Africa	2015	ERR6908282
Pf021_HKCCLCCXY_L3	Mfangano	Kenya_West	Kenya	East Africa	2015	ERR6908283
Pf023_HK7MKCCXY_L3	Mfangano	Kenya_West	Kenya	East Africa	2015	ERR6908284
Pf024_HKCCLCCXY_L3	Mfangano	Kenya_West	Kenya	East Africa	2015	ERR6908285
Pf025_HKCCLCCXY_L3	Mfangano	Kenya_West	Kenya	East Africa	2015	ERR6908286
Pf026_HK7MKCCXY_L3	Mfangano	Kenya_West	Kenya	East Africa	2015	ERR6908287
Pf028_HKCCLCCXY_L3	Mfangano	Kenya_West	Kenya	East Africa	2015	ERR6908288
Pf029_HKCCLCCXY_L3	Mfangano	Kenya_West	Kenya	East Africa	2015	ERR6908289
QS0056-C	West Arsi Zone	—	Ethiopia	North East Africa	2013	ERR1035493
QS0087-C	West Arsi Zone	—	Ethiopia	North East Africa	2013	ERR1035520
QS0104-C	West Arsi Zone	—	Ethiopia	North East Africa	2013	ERR1035536
QS0109-C	West Arsi Zone	—	Ethiopia	North East Africa	2013	ERR1045266
QS0110-C	West Arsi Zone	—	Ethiopia	North East Africa	2013	ERR1045267
QS0127-C	West Arsi Zone	—	Ethiopia	North East Africa	2013	ERR1045281

Sample	District/Zone	Region	Country	African region	Collection year	Sequence accession
QS0129-C	West Arsi Zone	—	Ethiopia	North East Africa	2013	ERR1045283
QS0132-C	West Arsi Zone	—	Ethiopia	North East Africa	2013	ERR1045286
QS0135-C	West Arsi Zone	—	Ethiopia	North East Africa	2013	ERR1045288
QS0144-C	West Arsi Zone	—	Ethiopia	North East Africa	2013	ERR1045295
QS0154-C	Shewa Robit Town Health Centre	—	Ethiopia	North East Africa	2015	ERR1106575
QS0159-C	Shewa Robit Town Health Centre	—	Ethiopia	North East Africa	2015	ERR1106579
QS0162-C	Shewa Robit Town Health Centre	—	Ethiopia	North East Africa	2015	ERR1106581
QS0163-C	Shewa Robit Town Health Centre	—	Ethiopia	North East Africa	2015	ERR1106582
QS0169-C	Shewa Robit Town Health Centre	—	Ethiopia	North East Africa	2015	ERR1106587
QS0170-C	Shewa Robit Town Health Centre	—	Ethiopia	North East Africa	2015	ERR1106590
SPT00898	Kassala	—	Sudan	North East Africa	2015	ERR2299571
SPT00901	Kassala	—	Sudan	North East Africa	2015	ERR2299574
SPT00902	Kassala	—	Sudan	North East Africa	2015	ERR2299575
SPT38368	Khartoum	—	Sudan	North East Africa	2017	ERR3609483
SPT38388	Khartoum	—	Sudan	North East Africa	2017	ERR3609499
SPT38540	Khartoum	—	Sudan	North East Africa	2016	ERR3609275
SPT46076	Pwani	Tanzania_East	Tanzania	East Africa	2019	ERR4021329
SPT46094	Pwani	Tanzania_East	Tanzania	East Africa	2019	ERR4021335
SPT46112	Pwani	Tanzania_East	Tanzania	East Africa	2019	ERR4021297
SPT46113	Pwani	Tanzania_East	Tanzania	East Africa	2019	ERR4021288
SPT46135	Pwani	Tanzania_East	Tanzania	East Africa	2019	ERR4021091
SPT46152	Pwani	Tanzania_East	Tanzania	East Africa	2019	ERR4021344

Sample	District/Zone	Region	Country	African region	Collection year	Sequence accession
SPT46162	Pwani	Tanzania_East	Tanzania	East Africa	2019	ERR4021280
SPT46163	Pwani	Tanzania_East	Tanzania	East Africa	2019	ERR4021333
SPT46226	Pwani	Tanzania_East	Tanzania	East Africa	2019	ERR4020838
SPT46235	Pwani	Tanzania_East	Tanzania	East Africa	2019	ERR4021267
SPT46238	Pwani	Tanzania_East	Tanzania	East Africa	2019	ERR4021236
SPT46265	Pwani	Tanzania_East	Tanzania	East Africa	2019	ERR4020970
SPT46267	Pwani	Tanzania_East	Tanzania	East Africa	2019	ERR4021314
SPT46283	Pwani	Tanzania_East	Tanzania	East Africa	2019	ERR4021046
SPT46300	Pwani	Tanzania_East	Tanzania	East Africa	2019	ERR4021320
SPT46307	Pwani	Tanzania_East	Tanzania	East Africa	2019	ERR4021000
SPT46319	Pwani	Tanzania_East	Tanzania	East Africa	2019	ERR4021013
SPT46323	Pwani	Tanzania_East	Tanzania	East Africa	2019	ERR4020989
SPT46324	Pwani	Tanzania_East	Tanzania	East Africa	2019	ERR4020983
SPT46335	Pwani	Tanzania_East	Tanzania	East Africa	2019	ERR4021050
SPT46350	Pwani	Tanzania_East	Tanzania	East Africa	2019	ERR4021052
SPT46352	Pwani	Tanzania_East	Tanzania	East Africa	2019	ERR4021319
SPT46421	Pwani	Tanzania_East	Tanzania	East Africa	2019	ERR4021070
SPT46457	Pwani	Tanzania_East	Tanzania	East Africa	2019	ERR4288959
SPT46474	Pwani	Tanzania_East	Tanzania	East Africa	2019	ERR4021142
SPT46477	Pwani	Tanzania_East	Tanzania	East Africa	2019	ERR4021149
SPT46482	Pwani	Tanzania_East	Tanzania	East Africa	2019	ERR4021140
SPT46503	Pwani	Tanzania_East	Tanzania	East Africa	2019	ERR4021192
SPT46521	Pwani	Tanzania_East	Tanzania	East Africa	2019	ERR4021201
SPT46555	Pwani	Tanzania_East	Tanzania	East Africa	2019	ERR4288983
SPT53928	Kilifi	Kenya_East	Kenya	East Africa	2019	ERR5839394
SPT53932	Kilifi	Kenya_East	Kenya	East Africa	2019	ERR5839347
SPT53933	Kilifi	Kenya_East	Kenya	East Africa	2019	ERR5839378
SPT53936	Kilifi	Kenya_East	Kenya	East Africa	2019	ERR5839401
SPT53951	Kilifi	Kenya_East	Kenya	East Africa	2019	ERR5839369
SPT53952	Kilifi	Kenya_East	Kenya	East Africa	2019	ERR5839348
SPT53960	Kilifi	Kenya_East	Kenya	East Africa	2019	ERR5839523
SPT66893	Kilifi	Kenya_East	Kenya	East Africa	2019	ERR9515544
SPT66895	Kilifi	Kenya_East	Kenya	East Africa	2019	ERR9515546
SPT66898	Kilifi	Kenya_East	Kenya	East Africa	2019	ERR9515547
SPT66903	Kilifi	Kenya_East	Kenya	East Africa	2019	ERR9515613
SPT66920	Kilifi	Kenya_East	Kenya	East Africa	2019	ERR9515549
SPT66928	Kilifi	Kenya_East	Kenya	East Africa	2019	ERR9515551
SPT66939	Kilifi	Kenya_East	Kenya	East Africa	2019	ERR9515614
SPT66966	Kilifi	Kenya_East	Kenya	East Africa	2019	ERR9515629

Sample	District/Zone	Region	Country	African region	Collection year	Sequence accession
SPT66986	Kilifi	Kenya_East	Kenya	East Africa	2019	ERR9515570
SPT66987	Kilifi	Kenya_East	Kenya	East Africa	2019	ERR9515571
SPT67008	Kilifi	Kenya_East	Kenya	East Africa	2020	ERR9515583
SPT67010	Kilifi	Kenya_East	Kenya	East Africa	2020	ERR9515584
SPT67011	Kilifi	Kenya_East	Kenya	East Africa	2020	ERR9515585
SPT67012	Kilifi	Kenya_East	Kenya	East Africa	2020	ERR9515586
SPT67023	Kilifi	Kenya_East	Kenya	East Africa	2019	ERR9515541
SPT67058	Kilifi	Kenya_East	Kenya	East Africa	2019	ERR9515719
SPT67068	Kilifi	Kenya_East	Kenya	East Africa	2019	ERR9515723
SPT67073	Kilifi	Kenya_East	Kenya	East Africa	2019	ERR9515727
SPT67093	Kilifi	Kenya_East	Kenya	East Africa	2019	ERR9515652
SPT67104	Kilifi	Kenya_East	Kenya	East Africa	2019	ERR9515658
SPT67128	Kilifi	Kenya_East	Kenya	East Africa	2019	ERR9515677
SPT67134	Kilifi	Kenya_East	Kenya	East Africa	2019	ERR9515683
SPT67164	Kilifi	Kenya_East	Kenya	East Africa	2019	ERR9515733
SPT67170	Kilifi	Kenya_East	Kenya	East Africa	2019	ERR9515738
SPT67182	Kilifi	Kenya_East	Kenya	East Africa	2019	ERR9515747
SPT72025	Kinshasa	—	DRC	Central Africa	2022	ERR10685567
SPT72087	Kinshasa	—	DRC	Central Africa	2022	ERR10685613
SPT72089	Kinshasa	—	DRC	Central Africa	2022	ERR10685561
SPT72125	Kinshasa	—	DRC	Central Africa	2022	ERR10685534
SPT72191	Kinshasa	—	DRC	Central Africa	2022	ERR10685509
SPT72247	Kinshasa	—	DRC	Central Africa	2022	ERR10685393
SPT72252	Kinshasa	—	DRC	Central Africa	2022	ERR10685543
SPT72259	Kinshasa	—	DRC	Central Africa	2022	ERR10685389
SPT72271	Kinshasa	—	DRC	Central Africa	2022	ERR10685408
SPT72285	Kinshasa	—	DRC	Central Africa	2022	ERR10685383
SPT72286	Kinshasa	—	DRC	Central Africa	2022	ERR10685379
SPT72309	Kinshasa	—	DRC	Central Africa	2022	ERR10763977

Sample	District/Zone	Region	Country	African region	Collection year	Sequence accession
SPT72311	Kinshasa	—	DRC	Central Africa	2022	ERR10763968
SPT72328	Kinshasa	—	DRC	Central Africa	2022	ERR10763976
SPT72337	Kinshasa	—	DRC	Central Africa	2022	ERR10763954
SPT72340	Kinshasa	—	DRC	Central Africa	2022	ERR10763926
SPT72361	Kinshasa	—	DRC	Central Africa	2022	ERR10763961
SPT72367	Kinshasa	—	DRC	Central Africa	2022	ERR10763960
SPT72373	Kinshasa	—	DRC	Central Africa	2022	ERR10763930
SPT72389	Kinshasa	—	DRC	Central Africa	2022	ERR10763985
SPT72408	Kinshasa	—	DRC	Central Africa	2022	ERR10764010
SPT72418	Kinshasa	—	DRC	Central Africa	2022	ERR10764002
SPT72421	Kinshasa	—	DRC	Central Africa	2022	ERR10764007
SPT72427	Kinshasa	—	DRC	Central Africa	2022	ERR10763979
SPT87699	Kinshasa	—	DRC	Central Africa	2022	ERR11840555
SPT87702	Kinshasa	—	DRC	Central Africa	2022	ERR11840552
SPT87729	Kinshasa	—	DRC	Central Africa	2022	ERR11840541
SPT87737	Kinshasa	—	DRC	Central Africa	2022	ERR11840534
SPT87745	Kinshasa	—	DRC	Central Africa	2022	ERR11840571
SPT87977	Kinshasa	—	DRC	Central Africa	2022	ERR11840472
SPT87979	Kinshasa	—	DRC	Central Africa	2022	ERR11840470
SPT87986	Kinshasa	—	DRC	Central Africa	2022	ERR11840480
SPT87995	Kinshasa	—	DRC	Central Africa	2022	ERR11840502
SPT88010	Kinshasa	—	DRC	Central Africa	2022	ERR11840492

Sample	District/Zone	Region	Country	African region	Collection year	Sequence accession
SPT88018	Kinshasa	—	DRC	Central Africa	2022	ERR11840425
SPT88048	Kinshasa	—	DRC	Central Africa	2022	ERR11840439
SPT88145	Kinshasa	—	DRC	Central Africa	2022	ERR11840798
SPT88148	Kinshasa	—	DRC	Central Africa	2022	ERR11840801
SPT88181	Kinshasa	—	DRC	Central Africa	2022	ERR11840843
SPT88273	Kinshasa	—	DRC	Central Africa	2022	ERR11840835
SPT88310	Kinshasa	—	DRC	Central Africa	2022	ERR11840901
SPT88323	Kinshasa	—	DRC	Central Africa	2022	ERR11840904
SPT88327	Kinshasa	—	DRC	Central Africa	2022	ERR11840870
SPT88348	Kinshasa	—	DRC	Central Africa	2022	ERR11840882
SPT88393	Kinshasa	—	DRC	Central Africa	2022	ERR11840920
SPT88398	Kinshasa	—	DRC	Central Africa	2022	ERR11840876
SPT88452	Kinshasa	—	DRC	Central Africa	2022	ERR11840952
SPT88454	Kinshasa	—	DRC	Central Africa	2022	ERR11840951
SPT88514	Kinshasa	—	DRC	Central Africa	2022	ERR11871804
SPT88563	Kinshasa	—	DRC	Central Africa	2022	ERR11871853
SPT88578	Kinshasa	—	DRC	Central Africa	2022	ERR11871830
SPT88693	Kinshasa	—	DRC	Central Africa	2022	ERR11871721
SPT88715	Kinshasa	—	DRC	Central Africa	2022	ERR11871752
SPT88722	Kinshasa	—	DRC	Central Africa	2022	ERR11871759
SPT88723	Kinshasa	—	DRC	Central Africa	2022	ERR11871760
SPT88727	Kinshasa	—	DRC	Central Africa	2022	ERR11871775

Sample	District/Zone	Region	Country	African region	Collection year	Sequence accession
SPT88750	Kinshasa	—	DRC	Central Africa	2022	ERR11871781
SPT88755	Kinshasa	—	DRC	Central Africa	2022	ERR11871786
SPT88760	Kinshasa	—	DRC	Central Africa	2022	ERR11871771
T0907AD0	Karagwe	Tanzania_West	Tanzania	East Africa	2022	SRR29397016
T0909BD0	Karagwe	Tanzania_West	Tanzania	East Africa	2022	SRR29397011
T0911AD0	Karagwe	Tanzania_West	Tanzania	East Africa	2022	SRR29397007
T0913AD0	Karagwe	Tanzania_West	Tanzania	East Africa	2022	SRR29397003
T0918BD0	Karagwe	Tanzania_West	Tanzania	East Africa	2022	SRR29396972
T0919AD0	Karagwe	Tanzania_West	Tanzania	East Africa	2022	SRR29396971
T0919BD0	Karagwe	Tanzania_West	Tanzania	East Africa	2022	SRR29396970
T0922AD0	Karagwe	Tanzania_West	Tanzania	East Africa	2022	SRR29396964
T0922BD0	Karagwe	Tanzania_West	Tanzania	East Africa	2022	SRR29396963
T0923AD0	Karagwe	Tanzania_West	Tanzania	East Africa	2022	SRR29396962
T0925BD0	Karagwe	Tanzania_West	Tanzania	East Africa	2022	SRR29396998
T0926AD0	Karagwe	Tanzania_West	Tanzania	East Africa	2022	SRR29396997
T0926BD0	Karagwe	Tanzania_West	Tanzania	East Africa	2022	SRR29396995
T0928AD0	Karagwe	Tanzania_West	Tanzania	East Africa	2022	SRR29396992
T0930AD0	Karagwe	Tanzania_West	Tanzania	East Africa	2022	SRR29396990
T0932AD0	Karagwe	Tanzania_West	Tanzania	East Africa	2022	SRR29396987
T0937BD0	Karagwe	Tanzania_West	Tanzania	East Africa	2022	SRR29396982
T0938AD0	Karagwe	Tanzania_West	Tanzania	East Africa	2022	SRR29396961
T0943BD0	Karagwe	Tanzania_West	Tanzania	East Africa	2022	SRR29396958
T0944AD0	Karagwe	Tanzania_West	Tanzania	East Africa	2022	SRR29396957
T0945AD0	Karagwe	Tanzania_West	Tanzania	East Africa	2022	SRR29396956
T0946BD0	Karagwe	Tanzania_West	Tanzania	East Africa	2022	SRR29396955
T0949BD0	Karagwe	Tanzania_West	Tanzania	East Africa	2022	SRR29396953
T0955BD0	Karagwe	Tanzania_West	Tanzania	East Africa	2022	SRR29396951
T0956BD0	Karagwe	Tanzania_West	Tanzania	East Africa	2022	SRR29396950
T0961AD0	Karagwe	Tanzania_West	Tanzania	East Africa	2022	SRR29396948
T0962AD0	Karagwe	Tanzania_West	Tanzania	East Africa	2022	SRR29396947
T0967BD0	Karagwe	Tanzania_West	Tanzania	East Africa	2022	SRR29396944
T0968BD0	Karagwe	Tanzania_West	Tanzania	East Africa	2022	SRR29396942
T0973AD0	Karagwe	Tanzania_West	Tanzania	East Africa	2022	SRR29396941
T0980AD0	Karagwe	Tanzania_West	Tanzania	East Africa	2022	SRR29396939
T0983BD0	Karagwe	Tanzania_West	Tanzania	East Africa	2022	SRR29396936
T0987AD0	Karagwe	Tanzania_West	Tanzania	East Africa	2022	SRR29396934
UGK_707_3-SRX146743	Kampala	Uganda_East-Past	Uganda	East Africa	—	SRR641960

Sample	District/Zone	Region	Country	African region	Collection year	Sequence accession
Uganda-AG-04-11	Agago	Uganda_North-Past	Uganda	East Africa	2019	SRR34783618
Uganda-AG-04-13	Agago	Uganda_North-Past	Uganda	East Africa	2019	SRR34783538
Uganda-AG-05-15	Agago	Uganda_North-Past	Uganda	East Africa	2020	SRR34783511
Uganda-AG-05-22	Agago	Uganda_North-Past	Uganda	East Africa	2020	SRR34783500
Uganda-AG-05-41	Agago	Uganda_North-Past	Uganda	East Africa	2020	SRR34783595
Uganda-AG-05-47	Agago	Uganda_North-Past	Uganda	East Africa	2020	SRR34783584
Uganda-AG-05-49	Agago	Uganda_North-Past	Uganda	East Africa	2020	SRR34783562
Uganda-AG-05-56	Agago	Uganda_North-Past	Uganda	East Africa	2020	SRR34783553
Uganda-AG-05-59	Agago	Uganda_North-Past	Uganda	East Africa	2020	SRR34783552
Uganda-AG-05-81	Agago	Uganda_North-Past	Uganda	East Africa	2020	SRR34783548
Uganda-BUS-025	Mbale	Uganda_East-Past	Uganda	East Africa	2021	SRR34783542
Uganda-BUS-041	Mbale	Uganda_East-Past	Uganda	East Africa	2021	SRR34783540
Uganda-KBG-04-05	Kaabong	Uganda_North-East-Past	Uganda	East Africa	2019	SRR34783536
Uganda-KBG-05-65	Kaabong	Uganda_North-East-Past	Uganda	East Africa	2020	SRR34783482
Uganda-KBG-05-73	Kaabong	Uganda_North-East-Past	Uganda	East Africa	2020	SRR34783480
Uganda-KBG-05-84	Kaabong	Uganda_North-East-Past	Uganda	East Africa	2020	SRR34783478
Uganda-KO-05-14	Kole	Uganda_North-Past	Uganda	East Africa	2020	SRR34783477
Uganda-KO-05-75	Kole	Uganda_North-Past	Uganda	East Africa	2020	SRR34783471
Uganda-KTK-04-27	Katakwi	Uganda_North-East-Past	Uganda	East Africa	2019	SRR34783466
Uganda-KTK-04-45	Katakwi	Uganda_North-East-Past	Uganda	East Africa	2019	SRR34783464
Uganda-KTK-05-20	Katakwi	Uganda_North-East-Past	Uganda	East Africa	2020	SRR34783462
Uganda-KTK-05-21	Katakwi	Uganda_North-East-Past	Uganda	East Africa	2020	SRR34783535
Uganda-KTK-05-22	Katakwi	Uganda_North-East-Past	Uganda	East Africa	2020	SRR34783534
Uganda-KTK-05-45	Katakwi	Uganda_North-East-Past	Uganda	East Africa	2020	SRR34783531
Uganda-KTK-05-55	Katakwi	Uganda_North-East-Past	Uganda	East Africa	2020	SRR34783529
Uganda-KTK-05-56	Katakwi	Uganda_North-East-Past	Uganda	East Africa	2020	SRR34783528
Uganda-KTK-05-77	Katakwi	Uganda_North-East-Past	Uganda	East Africa	2020	SRR34783526
Uganda-KTK-05-79	Katakwi	Uganda_North-East-Past	Uganda	East Africa	2020	SRR34783525

Sample	District/Zone	Region	Country	African region	Collection year	Sequence accession
Uganda-KTK-05-97	Katakwi	Uganda_North-East-Past	Uganda	East Africa	2020	SRR34783524
Uganda-LA-02-47	Lamwo	Uganda_North-Past	Uganda	East Africa	2017	SRR34783516
Uganda-LA-05-10	Lamwo	Uganda_North-Past	Uganda	East Africa	2020	SRR34783510
Uganda-LA-05-17	Lamwo	Uganda_North-Past	Uganda	East Africa	2020	SRR34783509
Uganda-LA-05-25	Lamwo	Uganda_North-Past	Uganda	East Africa	2020	SRR34783507
Uganda-LA-05-30	Lamwo	Uganda_North-Past	Uganda	East Africa	2020	SRR34783506
Uganda-LA-05-39	Lamwo	Uganda_North-Past	Uganda	East Africa	2020	SRR34783504
Uganda-LA-05-52	Lamwo	Uganda_North-Past	Uganda	East Africa	2020	SRR34783502
Uganda-LA-05-59	Lamwo	Uganda_North-Past	Uganda	East Africa	2020	SRR34783497
Uganda-LA-05-66	Lamwo	Uganda_North-Past	Uganda	East Africa	2020	SRR34783496
Uganda-LA-05-75	Lamwo	Uganda_North-Past	Uganda	East Africa	2020	SRR34783493
Uganda-LA-05-79	Lamwo	Uganda_North-Past	Uganda	East Africa	2020	SRR34783492
Uganda-LA-05-89	Lamwo	Uganda_North-Past	Uganda	East Africa	2020	SRR34783489
Uganda-PAT-005-P2-D7	Agago	Uganda_North-Past	Uganda	East Africa	2021	SRR34783609
Uganda-PAT-011-P2-A3	Agago	Uganda_North-Past	Uganda	East Africa	2021	SRR34783603
Uganda-PAT-015-P2-H4	Agago	Uganda_North-Past	Uganda	East Africa	2021	SRR34783600
Uganda-PAT-018	Agago	Uganda_North-Past	Uganda	East Africa	2021	SRR34783597
Uganda-PAT-019-P2-F6	Agago	Uganda_North-Past	Uganda	East Africa	2021	SRR34783596
Uganda-PAT-020-P2-H6	Agago	Uganda_North-Past	Uganda	East Africa	2021	SRR34783594
Uganda-PAT-024-P2-B10	Agago	Uganda_North-Past	Uganda	East Africa	2021	SRR34783592
Uganda-PAT-039	Agago	Uganda_North-Past	Uganda	East Africa	2021	SRR34783585
Uganda-PAT-040	Agago	Uganda_North-Past	Uganda	East Africa	2021	SRR34783583
Uganda-PAT-043	Agago	Uganda_North-Past	Uganda	East Africa	2021	SRR34783582
Uganda-PAT-045	Agago	Uganda_North-Past	Uganda	East Africa	2021	SRR34783581
Uganda-PAT-046	Agago	Uganda_North-Past	Uganda	East Africa	2021	SRR34783580
Uganda-PAT-048	Agago	Uganda_North-Past	Uganda	East Africa	2021	SRR34783578
Uganda-PAT-052	Agago	Uganda_North-Past	Uganda	East Africa	2021	SRR34783577
Uganda-PAT-071	Agago	Uganda_North-Past	Uganda	East Africa	2022	SRR34783570
Uganda-PBC-261-12Feb21	Tororo	Uganda_East-Past	Uganda	East Africa	2021	SRR34783564
Uganda-TDH-173-P2-B11	Tororo	Uganda_East-Past	Uganda	East Africa	2021	SRR34783558
Uganda-TDH-174	Tororo	Uganda_East-Past	Uganda	East Africa	2021	SRR34783557
Uganda-TDH-190-P2-H2	Tororo	Uganda_East-Past	Uganda	East Africa	2021	SRR34783556
Uganda-TDH-191-P2-G8	Tororo	Uganda_East-Past	Uganda	East Africa	2021	SRR34783555
Uganda_Kampala1-SRX006181	Kampala	Uganda_East-Past	Uganda	East Africa	2006	SRR340211, SRR340216
Uganda_Kampala2-SRX006665	Kampala	Uganda_East-Past	Uganda	East Africa	2007	SRR340214, SRR340220
Uganda_Kampala3-SRX006734	Kampala	Uganda_East-Past	Uganda	East Africa	2006	SRR340224, SRR340225

Sample	District/Zone	Region	Country	African region	Collection year	Sequence accession
Uganda_Kampala4-SRX006178	Kampala	Uganda_East-Past	Uganda	East Africa	2006	SRR340208, SRR340221
Uganda_Kampala7-SRX007630	Kampala	Uganda_East-Past	Uganda	East Africa	2007	SRR340213, SRR340218
Uganda_Kampala9-SRX006735	Kampala	Uganda_East-Past	Uganda	East Africa	2007	SRR340215, SRR340226
CW_002_lib666283	Bungoma	Kenya_West	Kenya	East Africa	2018	ERR15534969
CW_003_lib666284	Bungoma	Kenya_West	Kenya	East Africa	2018	ERR15534970
CW_004_lib666285	Bungoma	Kenya_West	Kenya	East Africa	2018	ERR15534971
CW_006_lib666287	Bungoma	Kenya_West	Kenya	East Africa	2018	ERR15534972
CW_024_lib666305	Bungoma	Kenya_West	Kenya	East Africa	2018	ERR15534989
CW_030_lib666311	Bungoma	Kenya_West	Kenya	East Africa	2018	ERR15534995
CW_034_lib666315	Bungoma	Kenya_West	Kenya	East Africa	2018	ERR15534999
CW_042_lib666323	Bungoma	Kenya_West	Kenya	East Africa	2018	ERR15535007
CW_045_lib666326	Bungoma	Kenya_West	Kenya	East Africa	2018	ERR15535010
CW_046_lib666327	Bungoma	Kenya_West	Kenya	East Africa	2018	ERR15535011

Note: Metadata for external comparator samples and historical Ugandan samples used in the population-genetic analyses. Sample denotes the study sample identifier; District/Zone and region provide the available sub-national location or analytical grouping; African region and continent give broad geographic classification; collection year gives the year of sampling; and sequence accession is the public archive accession. An em dash indicates information that was not available in the source metadata. Groups ending in "-Past" denote historical Ugandan sample sets.

Supplementary Table 3. Country-wide distribution of alternative amino-acid alleles in pfpx1 (PF3D7_0720700) in Uganda

Gene	Gene ID	Reference AA	AA position	Alternative AA	Samples with allele, n	PIN-positive samples, n	Samples assessed at position, n	Allele prevalence	PIN-positive fraction	Variant ID
PX1	PF3D7_0720700	D	384	A	98	97	247	0.3968	0.9898	D384A
PX1	PF3D7_0720700	D	384	G	8	2	247	0.0324	0.2500	D384G
PX1	PF3D7_0720700	D	384	Y	2	2	247	0.0081	1.0000	D384Y
PX1	PF3D7_0720700	Y	1006	C	4	1	244	0.0164	0.2500	Y1006C
PX1	PF3D7_0720700	S	1211	R	16	0	244	0.0656	0.0000	S1211R
PX1	PF3D7_0720700	L	1222	P	177	166	244	0.7254	0.9379	L1222P
PX1	PF3D7_0720700	C	1484	F	2	0	236	0.0085	0.0000	C1484F
PX1	PF3D7_0720700	C	1484	Y	4	4	236	0.0169	1.0000	C1484Y
PX1	PF3D7_0720700	D	1502	E	3	0	236	0.0127	0.0000	D1502E
PX1	PF3D7_0720700	L	1570	V	7	0	236	0.0297	0.0000	L1570V
PX1	PF3D7_0720700	E	1591	K	4	0	236	0.0169	0.0000	E1591K
PX1	PF3D7_0720700	S	1673	N	211	160	236	0.8941	0.7583	S1673N
PX1	PF3D7_0720700	V	1686	M	2	0	236	0.0085	0.0000	V1686M
PX1	PF3D7_0720700	M	1701	I	166	160	236	0.7034	0.9639	M1701I
PX1	PF3D7_0720700	D	1705	N	166	160	236	0.7034	0.9639	D1705N
PX1	PF3D7_0720700	I	1707	M	3	0	236	0.0127	0.0000	I1707M
PX1	PF3D7_0720700	D	1711	N	36	0	236	0.1525	0.0000	D1711N
PX1	PF3D7_0720700	D	1889	G	4	2	239	0.0167	0.5000	D1889G
PX1	PF3D7_0720700	D	1889	N	3	3	239	0.0126	1.0000	D1889N
PX1	PF3D7_0720700	G	1891	K	2	2	238	0.0084	1.0000	G1891K
PX1	PF3D7_0720700	T	1903	A	5	0	236	0.0212	0.0000	T1903A
PX1	PF3D7_0720700	R	1951	I	5	0	236	0.0212	0.0000	R1951I
PX1	PF3D7_0720700	E	2160	K	3	0	249	0.0120	0.0000	E2160K

Note: Country-wide counts and proportions of non-synonymous alternative amino-acid alleles detected in PfPX11. Reference AA and AA position identify the reference residue and its protein position; Alternative AA is the observed replacement residue; Samples with allele is the number of samples carrying the alternative allele; PIN-positive samples is the number of allele-carrying samples classified as PIN-positive in the source analysis; Samples assessed at position is the denominator with callable data at that position; Allele prevalence = Samples with allele / Samples assessed at position; PIN-positive fraction = PIN-positive samples / Samples with allele; and Variant ID combines the reference residue, amino-acid position, and alternative residue. AA, amino acid; n, number of samples.
